# Numerical Simulation of Hydrogen Mixing Process in T-Junction Natural Gas Pipeline

**DOI:** 10.3390/ma18081879

**Published:** 2025-04-20

**Authors:** Yangyang Tian, Tongmu Tian, Gaofei Ren, Jiaxin Zhang

**Affiliations:** 1 College of Petroleum Engineering, Xi’an Shiyou University, Xi’an 710065, China; zhangjx200017@163.com; 2 Shaanxi Key Laboratory of Advanced Stimulation Technology for Oil & Gas Reservoirs, Xi’an 710065, China; 3 College of Mechanical and Transportation Engineering, China University of Petroleum, Beijing 102249, China; t2482161434@outlook.com; 4 China Petroleum Pipeline Engineering Corporation, Langfang 065000, China; 13061256562@163.com

**Keywords:** T-junction hydrogen-blended natural gas pipeline, numerical simulation, mixing length, mixing time

## Abstract

As a cost-effective transitional strategy, the integrated utilization and transportation of hydrogen and natural gas have gained significant attention as a viable pathway toward carbon neutrality. However, hydrogen’s low density, viscosity, and calorific value cause upward migration and accumulation in pipelines, raising embrittlement risks. Its high diffusion and leakage rates also pose significant safety challenges. To address hydrogen–natural gas blending challenges, achieving uniform mixing is crucial. This study systematically examines hydrogen–methane mixing in T-junction pipelines via numerical simulations, analyzing hydrogen mixing ratios (HMR: 10–25%) and methane flow rates (4–10 m/s) to assess flow and mixing dynamics. The coefficient of variation (COV) quantifies mixing uniformity with spatial and temporal analyses, optimizing hydrogen injection for rapid, homogeneous mixing. The key findings are as follows: (1) The uniform mixing length (the minimum axial distance required for the first pipeline cross-section to achieve 95% mixing uniformity) decreases inversely with the HMR, from 100 D to 20.875 D (D represents the pipeline diameter) as the HMR rises from 10% to 25%. (2) Analysis of initial uniform mixing time (defined as the duration required for the first pipeline cross-section to achieve 95% mixing uniformity) shows significant reduction with increasing HMR. While methane flow rate has a less pronounced effect, it nevertheless contributes to reducing the outlet uniform mixing time (defined as the time required to attain 95% mixing uniformity at the pipeline outlet). (3) A fundamental trade-off in engineering applications is established: increasing the HMR reduces mixing length but extends overall mixing time (difference between outlet and initial mixing times), while higher methane flow rates shorten overall mixing time at the cost of increased mixing length. The primary objective of this research is to elucidate the fundamental fluid dynamics of hydrogen–methane mixtures in T-junction pipelines, providing scientific insights for the safe and efficient operation of hydrogen-blended natural gas pipeline systems.

## 1. Introduction

As an ideal energy source for the 21st century, hydrogen energy is abundant in resources and emits no carbon during its utilization. Its adoption holds profound significance for China in achieving carbon peak and carbon neutrality, addressing global climate change and building a community with a shared future for mankind [1]. In recent years, to promote the transformation of China’s energy structure, reduce dependence on fossil fuels, and establish an environmentally friendly society, China has vigorously developed hydrogen energy. Many regions have incorporated the development of hydrogen energy into their 14th Five-Year Plans. Currently, the global demand for hydrogen is approximately 70 million tons per year (70 × 10^6^ t/a), with a total market value of about 125 billion USD (125 × 10^9^ USD).

The entire industry chain of hydrogen energy comprises three key stages: hydrogen production, hydrogen storage and transportation, and hydrogen utilization. Among these, large-scale, long-distance, and low-cost hydrogen storage and transportation represent the bottleneck restricting the development of hydrogen energy. To address this, some scholars have proposed utilizing existing natural gas pipelines for hydrogen transportation by blending hydrogen with natural gas. This approach avoids the challenges associated with newly constructed pure hydrogen pipelines, such as high initial investment costs, elevated operational expenses, and insufficient supporting infrastructure. Additionally, hydrogen can partially replace natural gas consumption, thereby reducing carbon emissions. Therefore, blending hydrogen into natural gas pipelines remains the optimal solution for hydrogen storage and transportation. However, the flow dynamics of hydrogen–natural gas mixtures in pipelines are not yet fully understood, making it imperative to conduct simulation research on the process of mixing hydrogen into natural gas pipelines.

Currently, natural gas–hydrogen blending pipeline projects remain limited globally but are undergoing a phase of rapid development. Selected operational and demonstration projects are summarized in Table 1. As indicated, publicly disclosed projects are sparse, with critical operational parameters (e.g., pressure, blending ratios) often lacking transparency. Existing projects universally adopt hydrogen blending ratios below 25%, which aligns with the upper threshold (25%) set in this study. Nevertheless, these pioneering efforts underscore the growing momentum and promising potential of hydrogen–natural gas co-transportation technologies in decarbonizing energy systems.

In terms of physicochemical property variations in hydrogen-blended natural gas systems, Li et al. [5] analyzed the impact of hydrogen on natural gas properties using the Soave–Benedict–Webb–Rubin (SBWR) equation of state (EOS). The study demonstrated that the isenthalpic curves rise with increasing hydrogen content, but the rate of ascent gradually diminishes. The Joule–Thomson coefficient was found to be more sensitive to hydrogen blending concentration at higher temperatures and lower pressures. Complementary research by Zhang Pu et al. [6] demonstrated that at 40% HMR, key parameters including higher heating value (HHV), relative density, Wobbe index, viscosity, and volumetric energy density (at 10 MPa, 20 °C) experienced reductions of approximately 27%, 35%, 10%, 20%, and 40%, respectively. Concurrently, compressibility factor and sonic velocity showed increases of 18% and 34% under identical conditions. Notably, current natural gas specifications impose limitations on permissible HMR ranges, while existing analytical protocols and equipment hydrogen tolerance thresholds remain inadequate for precise characterization of hydrogen–natural gas mixtures. Fiebig et al. [7] implemented the SmartSim pipeline network simulation tool to monitor gas quality parameters, particularly focusing on calorific value and compositional changes during hydrogen injection. In a separate investigation, Dell’Isola et al. [8] computed thermodynamic properties and compressibility factors for 25% HMR mixtures, identifying significant alterations in relative density, heat capacity, and HHV, along with HMR-dependent increases in compressibility. Experimental studies by Kobayashi et al. [9] utilized capillary viscometry quantified temperature/pressure-dependent viscosity characteristics of hydrogen–methane blends. Their measurements revealed non-linear viscosity reduction with increasing hydrogen content, with marked decreases becoming pronounced beyond 50% hydrogen concentration.

Regarding the safety of hydrogen-blended natural gas pipelines, Mahajan et al. [10] demonstrated that hydrogen exhibits a higher propensity to penetrate pipeline materials or eventually leak due to its significantly smaller molecular size compared to methane. This characteristic results in hydrogen leakage rates that may be 1.3 to 2.8 times higher than those of methane. Furthermore, the combustion limits of methane and hydrogen differ considerably, with methane having a range of 4–15% [11], while hydrogen exhibits a much broader range of 4–76% [12]. Shirvill et al. [13] reported that a hydrogen mole concentration below 25% does not generate significant explosion overpressure and thus does not substantially increase explosion risk. To assess the feasibility of hydrogen blending in natural gas pipelines, Cristello et al. [14] concluded that a hydrogen mole concentration of less than 20% does not pose a safety threat to natural gas pipeline integrity. However, as the hydrogen mole concentration increases, the maximum allowable working pressure of the pipeline may exceed its original design limits. Hafsi et al. [15] found that when the hydrogen mass ratio exceeded 2/3, the transient value of the maximum circumferential stress exceeded the allowable stress of pipeline steel X52. To prevent damage to the pipeline, they recommended limiting the hydrogen mass ratio to 30%. Subani et al. [16] proposed a technology based on the transient pressure wave of hydrogen–natural gas mixture, which was used to detect and locate leaks in rigid pipelines. Their experimental results demonstrated a significant dependence of detection performance on the hydrogen mass ratio (HMR) in the gas mixture. Specifically, under sudden valve closure conditions, the study revealed that increasing HMR led to corresponding enhancements in three key parameters: transient pressure, wave celerity, and mass flux. This phenomenon substantiates the theoretical correlation between hydrogen concentration and the enhanced combustion characteristics of the gas mixture.

Regarding the flow behavior in pipelines after hydrogen blending, Liu Cuiwei et al. [17] employed computational fluid dynamics (CFD) methods to demonstrate that at low flow rates, hydrogen tends to migrate toward the upper section of the pipeline, leading to the stratification of natural gas and hydrogen in hydrogen-blended natural gas pipelines. This stratification phenomenon is particularly pronounced under low-temperature and high-pressure conditions. Consequently, transporting hydrogen-blended natural gas at low pressure and high velocity is considered safer. Zhu Hongjun et al. [18] investigated the static stratification phenomenon in hydrogen-blended natural gas pipelines with undulating terrain during pipeline shutdown. They found that greater terrain relief, longer pipeline lengths, and larger pipeline volumes result in higher hydrogen volume fractions required to achieve stable stratification in the top horizontal section of the pipeline, thereby prolonging the time required to reach stable stratification. Yan Shuangjie et al. [19] proposed that the non-uniform mixing of natural gas and hydrogen is influenced by the composition of natural gas. Specifically, the higher concentrations of heavier hydrocarbons in natural gas, the longer the distance required for uniform mixing of natural gas and hydrogen. Zhang Heng et al. [20] established a mathematical model for transporting hydrogen-blended natural gas and found that the frictional resistance loss in the pipeline after mixing hydrogen is reduced, and the volumetric flow rate of gas mixture is increased. Since the calorific value of hydrogen is only 1/3 of that of natural gas, the calorific value of hydrogen-doped natural gas is lower than that of pure natural gas. The increase in volumetric flow rate is insufficient to compensate for the reduction in calorific value, resulting in a decrease in the energy flow rate of the pipeline and a reduction in gas transmission efficiency. Yan Shuangjie et al. [21] also constructed a three-dimensional model of a hydrogen- blended natural gas pipeline to study the effects of the hydrogen injection inlet structure and turbulence on the uniformity of natural gas and hydrogen mixing. The results showed that increasing the number of hydrogen injection inlets significantly reduces the distance required for uniform mixing of natural gas and hydrogen.

To predict the uniformity of hydrogen-blended natural gas, Suyue et al. [22] established a deep neural network (DNN) model, utilizing the coefficient of variation (COV) of hydrogen concentration to characterize the uniformity of hydrogen mixing. The average error of COV predicted by this model was only 4.53%, and the computational efficiency was also two orders of magnitude faster than that simulated by CFD. Wang Shuai et al. [23] established a T-junction hydrogen-blended natural gas pipeline. Their simulations using Simdroid and FLUENT revealed that increasing the hydrogen mixing ratio from 10% to 20% led to a significant rise in hydrogen concentration in the upper section of the pipeline, accompanied by more pronounced hydrogen stratification, with a width equivalent to one-third of the pipe diameter.

Yang Donghai et al. [24] conducted a numerical investigation on five types of static mixers. The study revealed that the gas mixing uniformity increases with both velocity and hydrogen blending ratio. Concurrently, the intensification of turbulence induced by increased flow velocity enhances mass transfer and consequently improves mixing uniformity. Cadorin et al. [25] employed Ansys CFX for finite element analysis of high-pressure gas flow in pipelines, discovering that hydrogen–methane mixtures (e.g., 90% methane and 10% hydrogen) under high Reynolds number conditions consume nearly twice as much energy as natural gas during transportation. This finding underscores the increased energy requirements for transporting hydrogen-blended natural gas. Tan et al. [26] further demonstrated that the extent of energy transportation cost depends on the volume fraction of hydrogen and flow conditions. Their analysis revealed that the cost of transporting pure hydrogen is minimized when the inlet flow rate approximates that of pure methane transportation, while the cost peaks when hydrogen is transported at the same mass flow rate as methane.

Bainier et al. [27] demonstrated that hydrogen injection into natural gas pipeline networks significantly reduces energy transmission efficiency. Their comparative analysis revealed that, under identical pressure ratios, the energy delivery decreased by 4%, 14%, and 15–20% for hydrogen mixing ratios of 10%, 40%, and 100%, respectively. Concurrently, the compressor energy input increased by 7%, 30%, and 210% for these respective ratios. This phenomenon is attributed to the combined effects of increased hydrogen content and high pressure drop, necessitating additional compressors to maintain energy supply within the gas network. In a related study, Cavana et al. [28] developed a gas network model that demonstrated that hydrogen injection into natural gas distribution networks could effectively eliminate gas quality variations in residential lines without compromising existing transmission infrastructure. Quintino et al. [29] further suggested that existing natural gas infrastructure could accommodate hydrogen with a volume fraction of 20% through minimal technical modifications. Gondal et al. [30] investigated the hydrogen tolerance of various pipeline network components, revealing significant variations across equipment types. Their findings indicated that compressors, as critical components of natural gas transmission systems, are limited to a maximum hydrogen mixing ratio of 10%. Conversely, distribution networks and gas storage equipment can accommodate up to 50% hydrogen mixing, while end-user equipment typically accepts ratios between 20% and 50%.

Tabkhi et al. [31] conducted a comprehensive assessment of hydrogen mixing impacts by setting the transmission power at 65% of the pipeline’s maximum capacity. Their analysis established a maximum permissible hydrogen mass ratio of 6.6%. Furthermore, they observed that maintaining constant energy flow in pipelines leads to increased energy consumption during transportation with higher hydrogen mixing ratios, necessitating additional compressor stations [25,27]. Wu et al. [32] numerically investigated the mixing characteristics of hydrogen injected into pipelines at multiple angles. The study found that increasing the hydrogen blending ratio enhances the kinetic energy of hydrogen, thereby reducing its penetration process into methane and avoiding local enrichment. Despite these advancements, the transient flow characteristics of T-junction hydrogen-blended natural gas pipelines remain insufficiently explored, highlighting a critical research gap in this field.

In summary, the development of hydrogen-blended natural gas pipelines faces three primary challenges: the absence of standardized technical protocols, elevated safety risks, and prohibitive capital costs, all of which significantly impede the advancement of hydrogen transportation infrastructure. These challenges fundamentally stem from an incomplete understanding of methane–hydrogen mixing dynamics within pipeline systems. Addressing the core issue—designing safe and cost-effective hydrogen–natural gas pipelines while ensuring homogeneous mixing—requires comprehensive elucidation of the interplay between hydrogen blending ratios (HBRs) and turbulence intensity on mixture uniformity.

Heterogeneous hydrogen–methane distribution over extended distances or durations poses dual risks: (1) localized hydrogen partial pressure surges may accelerate material degradation, compromising pipeline integrity; (2) fluctuations in calorific value and flowrate of blended gas could undermine metering accuracy and optimal hydrogen injection positioning. To establish robust technical standards for hydrogen–natural gas systems, systematic investigations into these factors are imperative.

In this study, we employ numerical simulations to investigate the transient flow characteristics of hydrogen-blended natural gas in a T-junction configuration. Our methodology rigorously examines the temporal evolution of (i) molar fraction distributions, (ii) mixing homogeneity, and (iii) pressure drop dynamics. Furthermore, we systematically elucidate the effects of hydrogen injection rates and methane flow rates on these critical parameters. The quantitative analysis of biphasic mixing behavior presented in this study provides valuable insights and practical references for the engineering implementation of hydrogen-blended natural gas systems.

## 2. Materials and Methods

### 2.1. Fundamental Assumptions

The numerical simulation is performed using ANSYS Fluent 2023R2, a commercial CFD software package. To simplify the model and enhance computational efficiency while maintaining result accuracy, the following assumptions were made for the hydrogen mixing process in the T-junction hydrogen-blended natural gas pipeline investigated in this study:

(1) Natural gas is approximated as pure methane (CH_4_).

**Figure 1:**
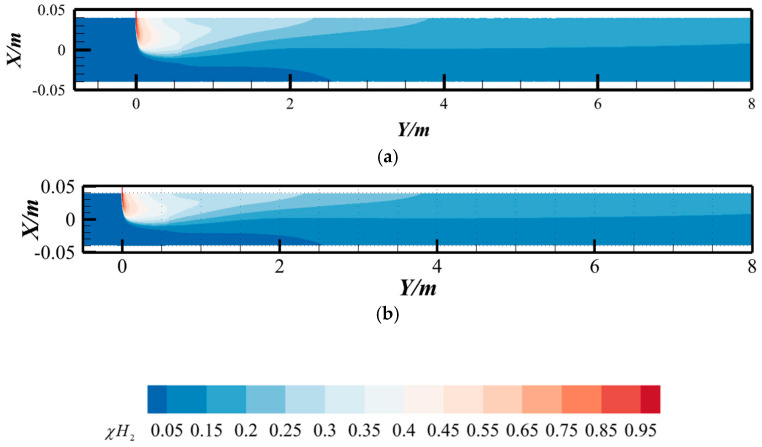
Comparison contours of hydrogen molar concentration with pure methane gas and real natural gas composition. (**a**) Real natural gas composition; (**b**) pure methane gas.

To validate this hypothesis, numerical simulations were performed using the natural gas composition reported by Uilhoorn [33] (molar fractions: CH_4_ = 98.3455%, C_2_H_6_ = 0.6104%, C_3_H_8_ = 0.1572%, n-C_4_H_10_ = 0.0299%, i-C_4_H_10_ = 0.0253%, n-C_5_H_12_= 0.0055%, i-C_5_H_12_= 0.0040%, N_2 =_ 0.0303%, CO_2_ = 0.7918%) under hydrogen-blended pipeline conditions. The hydrogen molar concentration contours at the *Z* = 0 cross-section were systematically compared with those obtained for pure methane gas under identical hydrogen blending scenarios, as shown in Figure 1. The results demonstrate minimal discrepancies between the two gas compositions, indicating negligible sensitivity of hydrogen dispersion patterns to the inclusion of minor hydrocarbon impurities under the studied conditions. But for natural gas with very high heavy hydrocarbon content, the mixing effect of hydrogen and natural gas will slightly weaken [21]. To rationalize the numerical modeling framework, this study exclusively considers natural gas with trace heavy hydrocarbon content. Consequently, the simplification of natural gas composition to pure methane represents a justified assumption, as the negligible concentration of heavy hydrocarbons (C^2+^components) ensures minimal impact on the mixing phenomena under the investigated operational conditions.

(2) Both methane and hydrogen are treated as incompressible gases, maintaining constant density throughout the flow process.

The compressibility of a gas is typically assessed by calculating its Mach number (*Ma*), where flows with *Ma* < 0.3 are considered incompressible due to negligible density variations, while *Ma* ≥ 0.3 necessitates compressibility considerations. Under the present study’s conditions—inlet temperature *T* = 288 K, methane inlet velocity *v_2_* = 4 m/s, and specific heat ratio *γ* ≈ 1.3 for the hydrogen–methane mixture—the Mach number is computed as follows:

(1) $$Ma=\frac{v_{2}}{\sqrt{\gamma RT}}=4/430\approx 0.0093\ll 0.3$$

From a Mach number perspective, gas compressibility effects can be considered negligible under the studied conditions (Ma < 0.3). However, this assumption necessitates further validation by analyzing the pressure gradient magnitude, as compressibility may still influence flow dynamics when local pressure variations exceed critical thresholds.

The compressibility criterion must also account for the relative pressure variation (Δ*P*/*P*_inlet_). Compressibility-induced density changes become non-negligible when Δ*P*/*P*_inlet_ > 10%, even at low Mach numbers.

At elevated pipeline pressures, the following occurs: (1) Density-driven effects: Increased gas density amplifies density gradients, altering the balance between convective transport and molecular diffusion. Concurrently, higher Reynolds numbers (Re = *ρvD*/*μ*) enhance turbulent mixing through intensified eddy diffusion. (2) Diffusion suppression: According to the Chapman–Enskog theory, the binary diffusion coefficient decreases with pressure. (3) Shock-induced interfacial disruption: At high flow velocities (*v*→*c*, where c is the local speed of sound), steep pressure gradients may trigger shock waves, generating discontinuous density/temperature jumps that destabilize the hydrogen–methane interface and impede homogeneity.

To rigorously evaluate compressibility’s influence, comparative simulations were conducted with and without the density-based model under identical boundary conditions. Following methodologies from analogous studies on T-junction hydrogen–methane mixing (e.g., Khabbazi et al. [34], Uilhoorn et al. [33]), the density model employed the Soave–Redlich–Kwong (SRK) equation of state, while preserving all other geometric and flow parameters.

**Figure 2:**
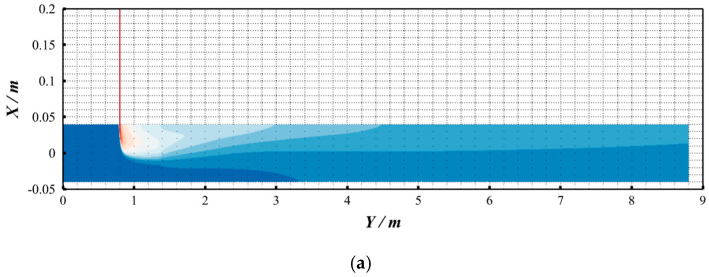

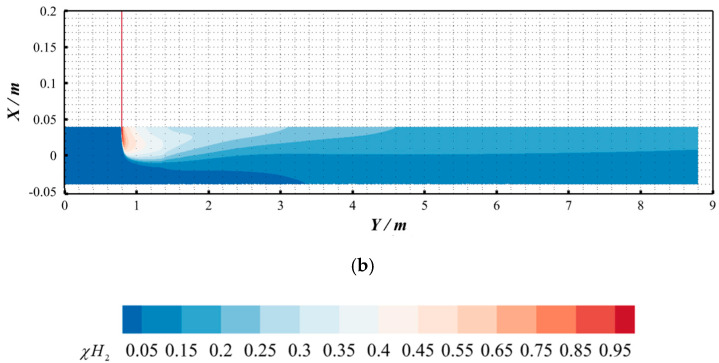
Comparison contours of hydrogen molar concentration in Case 2 at 3 s. (**a**) Compressible; (**b**) incompressible.

**Figure 3:**
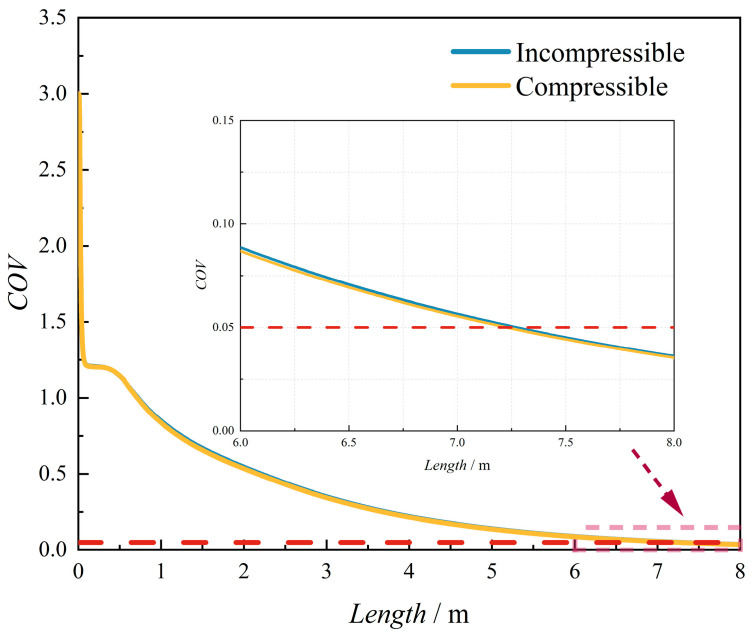
Comparison contours of hydrogen molar concentration in Case 2 at 3 s.

As shown in Figure 2, the hydrogen molar fraction distributions exhibit negligible discrepancies between compressible and incompressible models at the investigated pressure range, with identical spatial patterns and quantitative values. To quantify this observation, the coefficient of variation (COV)—a uniformity metric detailed in Section 2.10—was analyzed along the pipeline length (Figure 3). A pink dashed box (denoted by the red arrow) highlights the locally enlarged area to provide detailed structural visualization. The variations in the coefficient of variation (COV) along the pipeline length are highly consistent. This also indicates that, under the conditions of this study (temperature of 288 K and pipeline pressure of 3.5 MPa), considering compressibility has little influence on the hydrogen–methane mixing process. This finding is also consistent with the results of Gondal [30].

### 2.2. Governing Equations

The gas flow dynamics in a T-junction hydrogen-blended natural gas pipeline are governed by three fundamental conservation equations: the continuity equation (mass conservation), momentum conservation equation, and energy conservation equation. To accurately characterize the component distribution during hydrogen mixing and ensure the closure of the governing equation system, the species transport equation and turbulence model are additionally incorporated.

**Figure 4:**
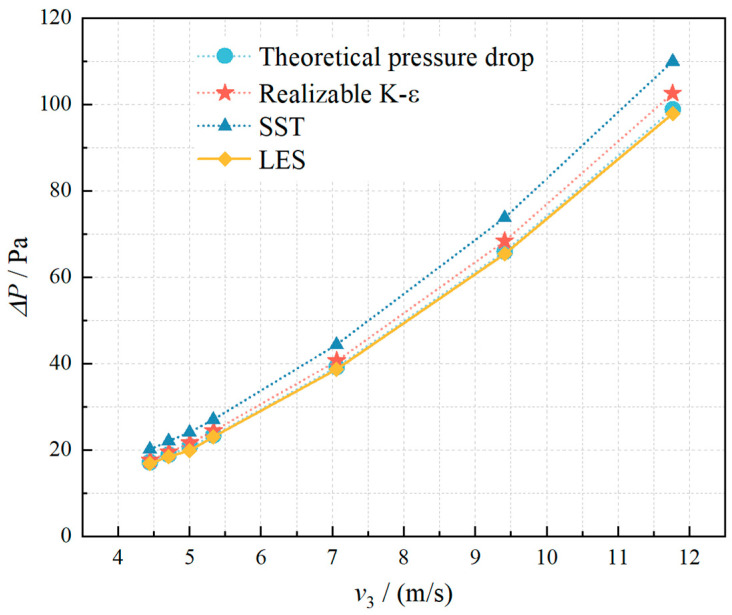
Comparison diagram of simulated and theoretical pressure drop values.

The selection of this turbulence model is justified by the similarity between the hydrogen injection process in the T-junction natural gas hydrogen-mixing pipeline and a circular jet flow. Kong Mingmin et al. [35] confirmed that the simulation results are consistent with those generated by the realizable k-epsilon turbulence model. Furthermore, a comparative analysis of pressure drop values under varying flow velocities was conducted using three turbulence models: the Large Eddy Simulation (LES) with the WALE subgrid-scale model, the Realizable k-ε model, and the k-ω SST model. The simulated results were validated against theoretical pressure drop predictions, as illustrated in Figure 4 (The theoretical pressure drop depicted in Figure is discussed in detail in Section 2.9, where the underlying principles are analytically derived). The data reveal that all three models exhibit close agreement in pressure drop calculations, with maximum deviations of 18.143% observed for the k-ω SST model. While the LES model demonstrated the smallest overall error margin, the Realizable k-ε model was ultimately selected for numerical model implementation to optimize the balance between computational accuracy and transient simulation time costs.

Continuity equation (mass conservation equation):

(2) $$\frac{\partial \rho }{\partial t}+\frac{\partial (\rho v_{x})}{\partial x}+\frac{\partial (\rho v_{y})}{\partial y}+\frac{\partial ({\rho v}_{z})}{\partial z}=0$$

where ρ represents the density of fluid; *t* denotes the flow time; and $v_{i}$ signifies the velocity in the direction *i*-direction.

Momentum conservation equation:

(3) $$\left\{\begin{array}{l} \frac{\partial (\rho v_{x})}{\partial t}+div(\rho v_{x}\vec{v})=div(\mu gradv_{x})-\frac{\partial p}{\partial x}+\rho F_{x}\\ \frac{\partial (\rho v_{y})}{\partial t}+div(\rho v_{y}\vec{v})=div(\mu gradv_{y})-\frac{\partial p}{\partial y}+\rho F_{y}\\ \frac{\partial (\rho v_{z})}{\partial t}+div(\rho v_{z}\vec{v})=div(\mu gradv_{z})-\frac{\partial p}{\partial z}+\rho F_{z} \end{array}\right.$$

where *p* signifies the pressure and *F*_j_ represents the mass force per unit mass acting in the *j*-direction.

Energy conservation equation:

(4) $$\frac{\partial (\rho T)}{\partial t}+div(\rho vT)=div(\frac{k}{c_{p}}gradT)+\frac{S_{T}}{c_{p}})$$

where *k* represents the heat transfer coefficient of gas; *c*_p_ denotes the specific heat capacity of the gas mixture; *T* represents the temperature; and *S_T_* indicates the volumetric heat source term within the fluid.

Component transport equation:

(5) $$\frac{\partial (\rho Y_{i})}{\partial t}+\nabla (\rho \vec{v}Y_{i})=-\nabla \vec{J_{i}}+R_{i}+S_{i}$$

where *Y_i_* denotes the mass fraction of component *i*; *R_i_* represents the net production rate of the chemical reaction; *S*_i_ signifies the production rate arising from discrete phase contributions and user-defined source terms; and Ji indicates the diffusion flux of component *i*.

(6) $$J_{i}=-D_{i}gradY_{i}$$

where *D*_i_ represents the diffusion coefficient.

Realizable k-epsilon turbulence equation:

(7) $$\frac{\partial (\rho k)}{\partial t}+\frac{\partial (\rho \vec{v_{i}}T)}{\partial x_{i}}=\frac{\partial }{\partial x_{i}}\left[\left(\mu +\frac{\mu_{t}}{\sigma_{k}}\right)\frac{\partial k}{\partial x_{i}}\right]+G_{k}+G_{b}+\rho \varepsilon -Y_{M}$$

(8) $$\frac{\partial (\rho \varepsilon )}{\partial t}+\frac{\partial (\rho \varepsilon \vec{v_{i}})}{\partial x_{i}}=\frac{\partial }{\partial x}\left[\left(\mu +\frac{\mu_{t}}{\sigma_{\varepsilon }}\right)\frac{\partial \varepsilon }{\partial x_{i}}\right]+\rho C_{1}S\varepsilon -\rho C_{2}\frac{\varepsilon^{2}}{k+\sqrt{v}\varepsilon }+C_{1\varepsilon }\frac{\varepsilon }{k}C_{3\varepsilon }G_{b}$$

(9) $$C_{1}=\max\left[0.43,\frac{\eta }{\eta +5}\right]$$

(10) $$\eta =S\frac{k}{\varepsilon }$$

(11) $$S=\sqrt{2S_{ij}S_{ij}}$$

(12) $$S_{ij}=\frac{1}{2}\left(\frac{\partial \vec{v_{i}}}{\partial x_{j}}+\frac{\partial \vec{v_{j}}}{\partial x_{i}}\right)$$

where *k* represents turbulence kinetic energy; *ε* denotes the turbulent kinetic energy dissipation rate; *u*_t_ signifies the eddy viscosity coefficient; *Y_M_* indicates the contribution of expansion in compressible turbulence to the total dissipation rate; *G_k_* represents the turbulent kinetic energy of the average velocity; *G_b_* denotes the turbulent kinetic energy generated by buoyancy; C_1*ε*_, C_2*ε*_, C_3*ε*_, *σ_k_* signifies the constant, respectively, taken as 1.22, 1.90, 0.09, 1.0; *σ_ε_* indicates the Prandtl number of the turbulent kinetic energy dissipation rate, taken as 1.2; $\vec{v_{i}}$ represents the velocity vector; *x_i_* signifies the position vector; and *S_ij_* represents the strain rate tensor.

### 2.3. Geometric Model

**Figure 5:**
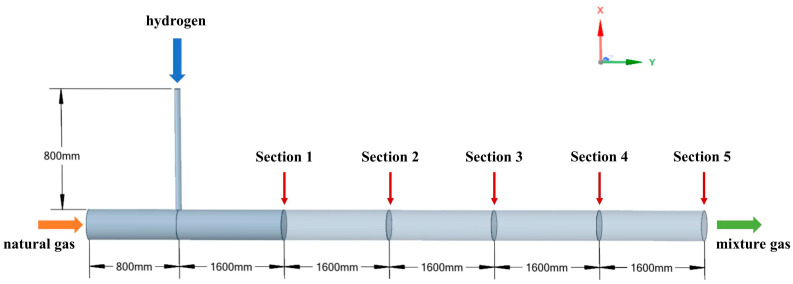
Schematic diagram of T-junction hydrogen-methane blending pipeline system.

The geometric configuration of the T-junction hydrogen-blended natural gas pipeline system is established in this study, as illustrated in Figure 5. The system comprises a methane transmission main pipeline (*D* = 80 mm) and a perpendicular hydrogen injection branch positioned 800 mm (10 *D*) downstream from the main inlet. The injection branch diameter was set at d = 0.2 *D* (16 mm) based on typical industrial blending ratios. Downstream of the injection point, the pipeline extends 8000 mm (100 *D*) to ensure a fully developed flow profile, incorporating five monitoring cross-sections spaced equidistantly at 1600 mm (20 *D*) intervals for boundary layer analysis. Methane was supplied through the upstream section while hydrogen was injected vertically via the side branch, enabling mixture formation governed by the Reynolds number (Re = *ρVD*/*μ*). The physical properties of methane and hydrogen transported by the T-junction natural gas hydrogen-mixing pipeline are summarized in Table 2.

### 2.4. Boundary Conditions

The computational domain employs four boundary types: (a) mass flow inlet boundaries for methane supply (main pipe) and hydrogen injection (branch pipe), (b) pressure outlet boundary with outflow condition, (c) no-slip walls under isothermal conditions (*T*_w_ = 288.15 K), and (d) Species transport model defining methane mass fraction as 1.0 at main inlet and 0.0 at the hydrogen branch. The methane flow rate values were selected based on the engineering specification for the design of a hydrogen transmission pipeline [36], which recommends a maximum allowable flow velocity of 20 m/s for pipeline systems. However, considering the relatively small diameter of the simulated pipeline in this study, we adopted a more conservative maximum methane flow velocity of 10 m/s to ensure operational safety and alignment with empirical guidelines. Operating parameter settings are shown in Table 3.

### 2.5. Mesh Strategy

**Figure 6:**
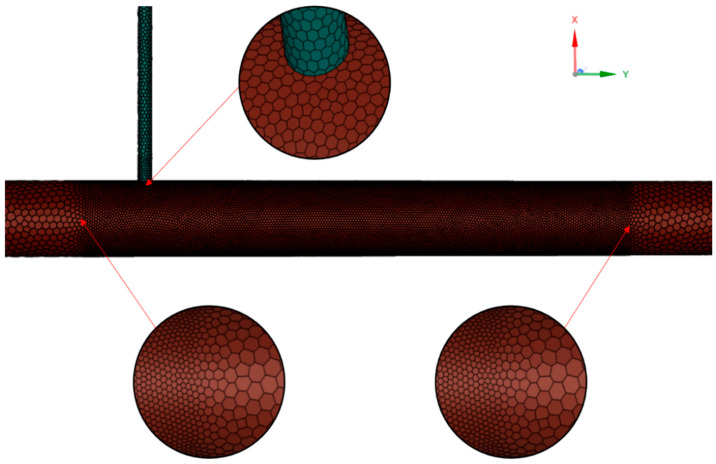
Schematic diagram of meshing.

The computational meshing methodology critically impacts numerical accuracy and convergence behavior. This study employs a hybrid Poly-Hexcore meshing based on “Mosaic” technology, strategically combining hexahedral-dominated core regions with polyhedral transitional zones. Three key meshing principles were implemented: (1) The Mosaic-enabled Poly-Hexcore topology achieves seamless node matching between hexahedral and polyhedral domains (As shown in the enlarged view above the pipeline in Figure 6), eliminating manual interface treatment and reducing total cell count by 38% compared to conventional hex-dominant meshing; (2) Local mesh refinement at the T-junction confluence between the main pipe and the branch pipe (minimum cell size = 0.02 *D*, growth rate = 1.2), as shown in the enlarged view below the pipeline in Figure 6; (3) The boundary layer mesh near the wall is also processed accordingly, and the scalable wall function is matched. This methodology neglects explicit resolution of the viscous sublayer and buffer layer, instead determining the first grid layer height based on an appropriate y+ value to ensure computational efficiency while preserving accuracy. Prism layers with (y^+^ < 15) satisfy the viscous sublayer requirements of the realizable *k*-*ε* turbulence model, while maintaining orthogonal quality greater than 0.98 for 95% of cells. A near-wall treatment approach was employed to model the boundary layer, utilizing wall functions to approximate near-wall flow behavior.

**Figure 7:**
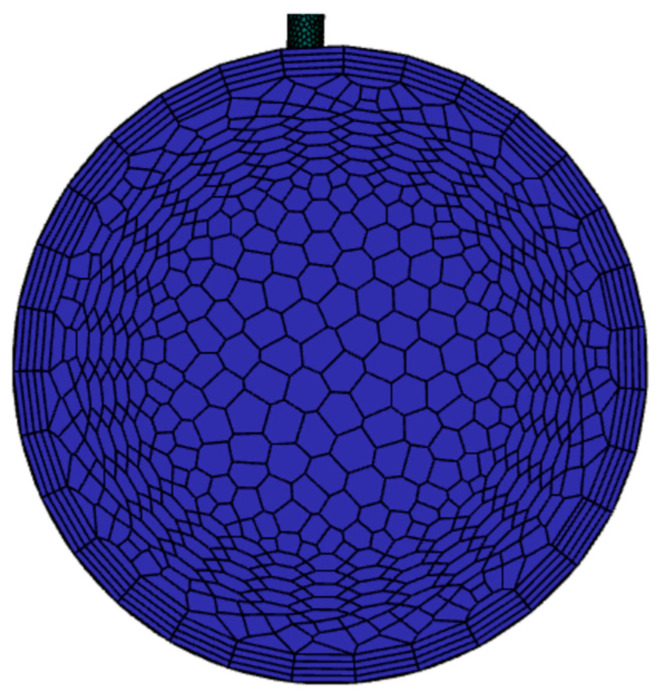
The details of the boundary layer.

To validate the rationality of the selected y+ range, first-layer grid height, and wall-function choice, simulations were conducted across seven operational conditions with mainstream velocities ranging from 4.44 m/s to 11.76 m/s. The first boundary layer grid height was set to 0.0013224776 m, corresponding to y+ values spanning 10–25 in the simulated results. This wide y+ range aligns with the applicability criteria of scalable wall functions, which are designed to handle moderate-to-high y+ regimes (typically y+ > 11) without requiring viscous sublayer resolution. The consistency of predicted y+ values across varying flow velocities confirms the robustness of the selected grid strategy and wall-function implementation. The boundary layer details are shown in Figure 7.

### 2.6. Grid Independence Verification

Computational accuracy exhibits positive correlation with mesh density, while incurring significant computational resource demands. To optimize this trade-off, grid independence analysis was conducted with three systematically refined meshes: (1) fine mesh: 888,781 cells (baseline); (2) medium mesh: 752,910 cells (25% coarsening); and (3) coarse mesh: 617,172 cells (44% coarsening).

The outlet section velocity of the three meshes were statistically analyzed. The results showed that the relative error between the simulated values of the outlet average velocity was from 0.83% to 1.89%, respectively, from fine to coarse, which met the accuracy requirements.

**Figure 8:**
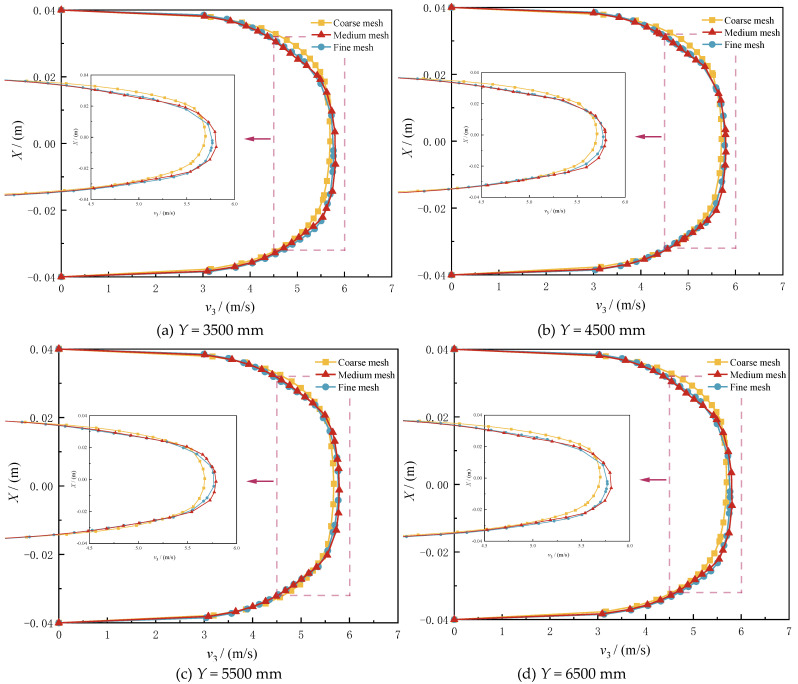
Velocity profile comparison of refined meshes (coarse/medium/fine) under turbulent flow conditions (Re = 20,466.83).

Axial velocity profiles at four transverse planes (Y = 43.75 *D*, 56.25 *D*, 68.75 *D*, 81.25 *D*) were comparatively analyzed (Figure 8). The medium-fine mesh comparison revealed less than 1.5% maximum velocity discrepancy, confirming spatial discretization adequacy. Consequently, the medium mesh was adopted for subsequent simulations, achieving 18.4% computational efficiency gain over the fine mesh while maintaining a less than 2% solution error.

### 2.7. Temporal Discretization Criteria

For transient numerical simulations, the selection of an optimal time step is critical to ensure numerical stability while minimizing computational cost. This study employs the Courant–Friedrichs–Lewy (CFL) condition to determine the maximum allowable time step:

(13) $$\Delta t=\frac{courant\times \Delta x}{u}$$

where courant take is 0 < courant < 1; Δ*x* denotes the grid spacing; and *u* represents the fluid flow velocity.

This stability criterion essentially constrains the number of grid cells that fluid can traverse within a single time step. An excessively large time step would lead to insufficient spatial resolution to resolve critical flow features, whereas an overly small time step would impose prohibitive computational costs. In accordance with the FLUENT Official User’s Guide, which recommends a CFL value of 1 for transient simulations, and consistent with prior studies on gas pipeline modeling where CFL values ≤ 1 are standard practice [37,38,39], we carefully balanced numerical stability and computational efficiency in this work. To ensure robustness while maintaining reasonable simulation times, a conservative CFL value of 0.8 was adopted. To balance numerical accuracy and computational efficiency, the time step in this study was dynamically determined through rigorous implementation of the CFL condition with a safety factor of 0.8.

### 2.8. Numerical Methodology

The computational framework fundamentally involves solving governing equations through iterative numerical schemes. Modern solvers provide multiple pressure–velocity coupling algorithms, including SIMPLE, SIMPLEC, PISO, and coupled methods. Strategic selection of these algorithms enhances convergence rates, optimizes computational efficiency, and prevents resource overutilization. Comparative studies evaluating SIMPLE, SIMPLEC, and PISO algorithms for lid-driven cavity flows demonstrate that while PISO exhibits superior robustness, it incurs higher computational overhead. Conversely, the SIMPLE algorithm achieves competitive convergence characteristics when paired with appropriate under-relaxation factors. Informed by these findings, the SIMPLE algorithm was employed in this study. The specific discretization strategies parameters are as follows:

- Pressure: Second-order central differencing;
- Momentum: Second-order upwind scheme;
- Turbulent kinetic energy: First-order upwind scheme;
- Turbulent dissipation rate: First-order upwind scheme;
- Species transport convection term: Second-order upwind scheme;
- Temporal discretization: First-order implicit formulation.

### 2.9. Model Validation

**Figure 9:**
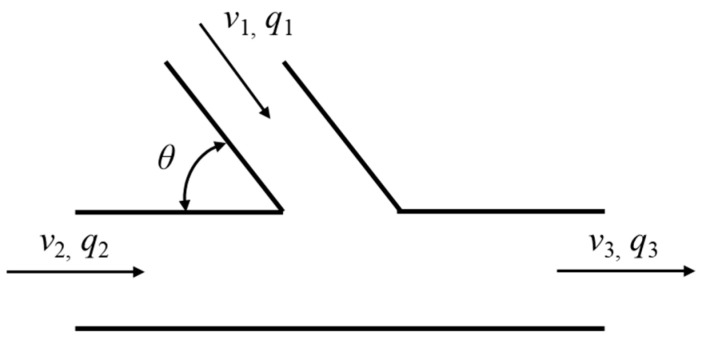
Combined flow in T-joint.

To validate the reliability of the T-junction hydrogen-blended natural gas pipeline model, the simulated pressure drop was benchmarked against theoretical predictions derived from the empirical pressure-loss coefficient correlation for T-junctions proposed by A. Gardel [40]. The pressure-loss coefficient (*K*_32_) for the T-junction (illustrated in Figure 9) is calculated using Gardel’s formula [40]:

(14) $$K_{32}=0.03(1-q^{2})-q^{2}\left[1+(1.62-r^{0.5})(\frac{\cos\theta }{a}-1)-0.38(1-a)\right]+(2-a)(1-q)q$$

where $a=\frac{A_{1}}{A_{3}}$, $q=\frac{q_{1}}{q_{3}}$.

Figure 4 illustrates the comparison between simulated and theoretical pressure drop values. The results demonstrate strong agreement between simulation outputs and theoretical calculations, with a maximum deviation of less than 4.5%. This confirms the validity and reliability of the adopted T-junction hydrogen–methane pipeline numerical model.

### 2.10. Evaluation of Mixing Uniformity

Various methodologies are currently available for assessing mixing uniformity. In this study, the coefficient of variation (COV), a dimensionless quantitative measure, was employed to evaluate the mixing homogeneity between methane and hydrogen. The COV is mathematically defined as the ratio of the standard deviation to the mean value of the sample volume fraction. The mathematical expression [41,42,43,44,45] is presented below:

(15) $$COV=\frac{1}{\bar{c}}\sqrt{\frac{\sum_{i=1}^{n}{\left(c_{i}-\bar{c}\right)}^{2}}{n-1}}$$

where *c_i_* represents the volume fraction of hydrogen gas at the sampling point; $\bar{c}$ denotes the average volume fraction of all samples; and *n* represents the total number of sample points.

The coefficient of variation (COV) ranges from 0 to 1. A higher COV value indicates greater non-uniformity in gas mixing, while a lower COV value corresponds to enhanced mixing uniformity. In engineering applications, a mixture is typically considered homogeneous when the COV is less than or equal to 0.05. Therefore, this criterion has been adopted in the present study.

Since the properties of hydrogen and methane calculated in this study closely approximate those of ideal gases, the mole fraction of hydrogen can be effectively equated to its volume fraction. Consequently, the volume fraction was calculated based on the mole fraction of hydrogen [46].

**Figure 10:**
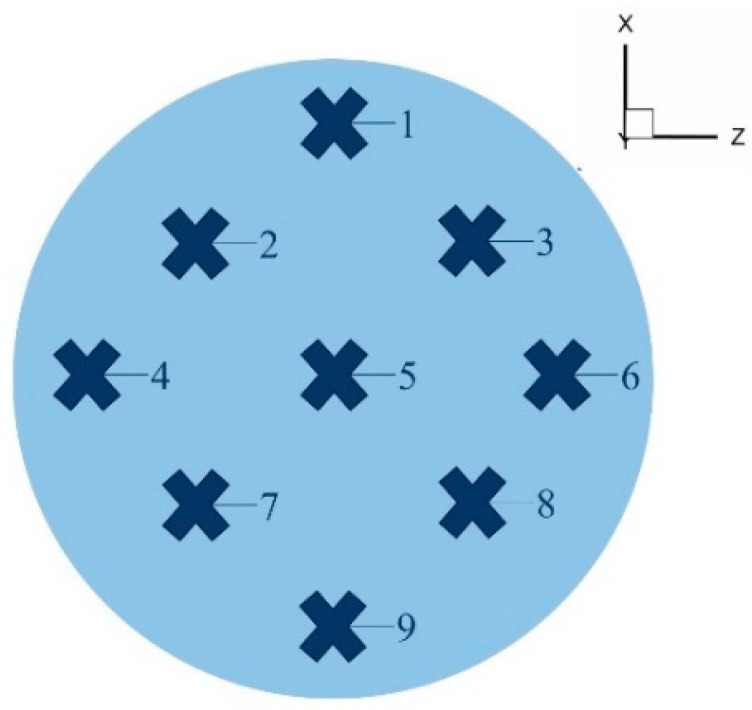
Schematic diagram of the monitoring point distribution in the cross-section.

To comprehensively investigate the hydrogen distribution, nine monitoring curves were established along the pipeline, extending from the hydrogen mixing point to the pipeline outlet section. Each cross-section of the pipeline was equipped with nine monitoring points, as illustrated in Figure 10, which provides a schematic representation of the monitoring point distribution. The precise coordinates of the monitoring curves are detailed in Table 4.

Based on the COV (coefficient of variation) values, this study proposes three quantitative parameters for assessing mixing homogeneity, which are defined as follows:

(1) Uniform mixing length: The minimum axial distance required for the first pipeline cross-section to achieve 95% mixing uniformity (COV ≤ 0.05). This parameter is determined by calculating the COV values along the entire pipeline length and plotting them as an XY-plot (abscissa: axial position Y along the flow direction; ordinate: COV values). The corresponding axial position at the first instance where COV ≤ 0.05 represents the uniform mixing length.

(2) Initial uniform mixing time: The time duration required for the first pipeline cross-section to reach 95% mixing uniformity. This is obtained by computing the temporal evolution of COV values along the pipeline and generating an XY-plot (abscissa: flow time; ordinate: COV values). The initial occurrence of COV ≤ 0.05 corresponds to the initial uniform mixing time.

(3) Overall mixing time: The temporal difference between outlet and initial mixing times. The outlet uniform mixing time is identified by analyzing the COV values at the pipeline outlet cross-section and determining the first instance of COV ≤ 0.05 in the XY-plot (abscissa: flow time; ordinate: COV values). The overall mixing time is the difference between outlet uniform mixing time and initial uniform mixing time.

These parameters provide a systematic framework for characterizing mixing performance in pipeline systems through time-resolved and spatially resolved COV analyses.

## 3. Results and Discussion

### 3.1. Flow Characteristics of Hydrogen–Methane Mixtures in Pipeline

To elucidate the flow characteristics in hydrogen–methane mixtures in the pipeline, a detailed analysis was conducted for Case 2 (as shown in Table 5, methane flow velocity: 4 m/s; hydrogen blending ratio: 15%), focusing on key parameters including hydrogen molar fraction, turbulent kinetic energy, and hydrogen partial pressure. Furthermore, a comprehensive study was conducted on the flow field properties under varying hydrogen mixing ratios (HMRs) and methane flow rates, aiming to better understand the mixing process when hydrogen is injected into natural gas pipelines.

This study primarily investigates the effects of HMR and methane flow rate on hydrogen mixing. A total of seven experimental groups (detailed in Table 4) were established: (1) Groups 1 to 4: The hydrogen mixing ratio (HMR) was increased from 10% to 25% while maintaining a constant methane flow rate. (2) Groups 2 and 5 to 7: The methane flow rate was increased from 4 m/s to 10 m/s while keeping the HMR constant.

#### 3.1.1. Turbulent Characteristics of the Mixture

**Figure 11:**
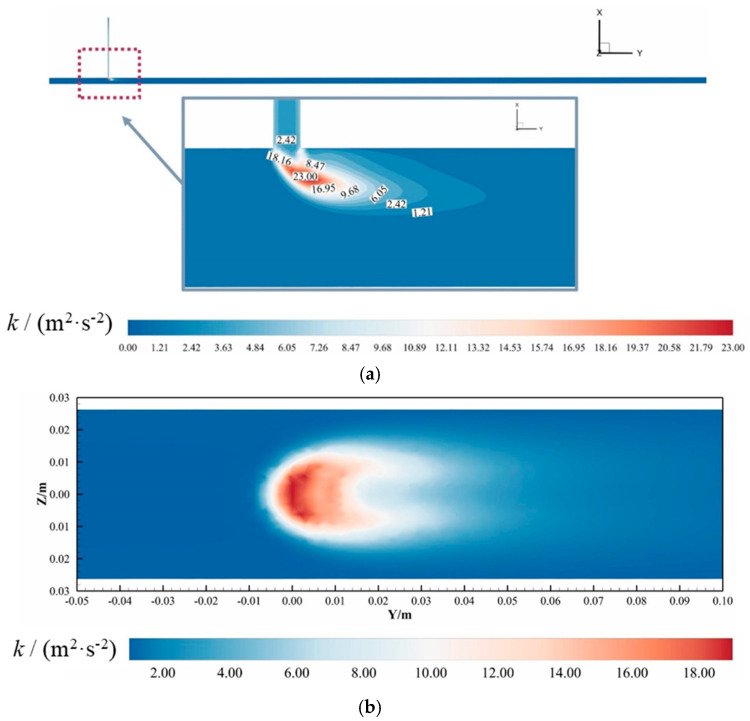
Distribution of turbulent kinetic energy in Case 2 at 3 s. (**a**) the global TKE profile at the Z = 0 m cross-section; (**b**) 0.5 D downstream of the injection point.

To systematically analyze turbulence characteristics in hydrogen-blended natural gas pipelines, Figure 11 presents the turbulent kinetic energy (TKE) distribution for Case 2 at 3 s. Panel (a) illustrates the global TKE profile at the *Z* = 0 m cross-section, while Panel (b) details localized TKE variations at *X* = 0.03 m. As shown in Figure 11a, the T-junction pipeline exhibits moderate turbulence levels (<1.21 m^2^/s^2^) across most regions, with a localized TKE surge (peak ≈ 23.00 m^2^/s^2^) occurring exclusively at the hydrogen injection junction—the interfacial zone where hydrogen and methane streams converge. Downstream of this junction, diminished turbulence levels (<0.85 m^2^/s^2^) correlate with prolonged mixing lengths and persistent stratification, as reduced turbulent diffusion impedes homogenization.

**Figure 12:**
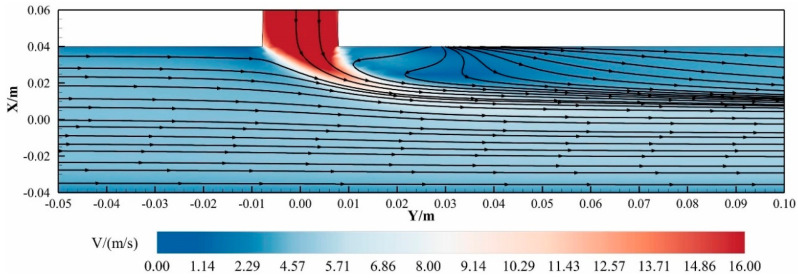
Streamline diagram of Z = 0 section in Case 2 at 3 s.

From Figure 11b, within 0.5 D downstream of the injection point, TKE intensifies abruptly (4.2–18.7 m^2^/s^2^) due to momentum exchange between the transverse hydrogen jet and axial methane flow. This turbulence amplification zone corresponds to the hydrodynamic shear layer formed at the interface. Beyond this region, TKE stabilizes at baseline levels (0.3–1.1 m^2^/s^2^), indicating limited penetration depth of the hydrogen jet. Streamline analysis (see supplementary flow trajectory, Figure 12. The arrow in the figure indicates the direction of flow) reveals that the hydrogen jet deflects parallel to the main pipeline axis at Y = 0.125 D, achieving a maximum penetration depth of 0.375 D. Consequently, lower pipeline regions remain unaffected by hydrogen injection, as evidenced by unaltered methane flow trajectories.

The restricted hydrogen penetration and turbulence decay stem from the jet’s insufficient momentum ratio relative to the methane mainstream. Buoyancy effects further exacerbate this limitation, as the low-density hydrogen stream preferentially migrates upward, creating a stratified shear layer. These combined factors—momentum deficit, buoyancy-driven stratification, and localized shear turbulence—govern the observed mixing inefficiency, with critical implications for pipeline material degradation due to prolonged hydrogen-rich zone exposure.

**Figure 13:**
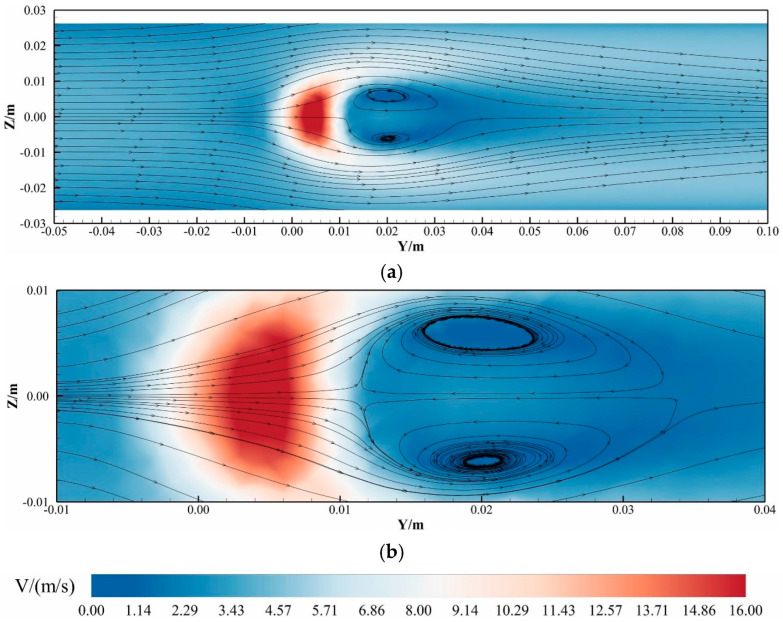
Streamline diagram of X = 0.03 m section in Case 2 at 3 s. (**a**) Streamline of hydrogen mixing point; (**b**) Localized enlarged of vortex.

To further investigate the hydrodynamic behavior within high-turbulence regions, the streamline topology at *X* = 0.03 m (0.375 *D*) was analyzed, as depicted in Figure 13 (The arrow in the figure indicates the direction of flow). This visualization elucidates the flow separation and vortex generation mechanisms arising from the collision between the methane mainstream and transverse hydrogen jet.

Dual Counter-Rotating Vortices: Two elliptically shaped, counter-rotating vortices form immediately downstream of the hydrogen injection point. These structures span 0.15 *D*–0.425 *D* in the axial direction (*Y*), with vortex cores centered at *Y* = 0.25 *D* and occupying a lateral width of 0.25 *D*.

Flow Constriction Effects: The hydrogen jet obstructs the pipeline’s cross-sectional area, inducing localized flow acceleration along both flanks of the jet due to the Bernoulli principle.

The vortex formation is attributed to shear layer instability at the hydrogen–methane interface, where the momentum ratio drives flow separation. The elliptical vortex geometry reflects energy dissipation patterns governed by viscous damping and turbulent diffusion. Furthermore, the accelerated flank flows enhance turbulent mixing within the shear layer but concurrently exacerbate localized wall shear stresses, presenting potential operational challenges for long-term pipeline integrity.

The aforementioned analysis reveals that hydrogen–methane mixing dynamics are governed by the interplay between buoyancy effects and turbulent diffusion. Insufficient turbulence intensity within pipelines promotes buoyancy-driven stratification and localized shear turbulence, resulting in prolonged exposure of pipeline materials to hydrogen-rich zones that critically degrade mechanical properties (e.g., the consequence of material changes due to hydrogen embrittlement, will occur over years [46]). Consequently, turbulence enhancement is paramount for ensuring safe hydrogen–natural gas pipeline operations. Previous studies have proposed strategies such as static mixers [41,42,43,44,45] and controlled wall roughness augmentation [47] to optimize mixing efficiency.

Notably, wall-roughness optimization assumes heightened importance in hydrogen-blended pipeline design compared to conventional natural gas systems. Increased surface roughness elevates the Darcy–Weisbach friction factor, amplifying near-wall shear stresses (Δτ ≈ 12–18 Pa per 10 μm roughness increment) and thereby augmenting turbulent kinetic energy production through enhanced velocity gradients. This mechanism intensifies turbulent fluctuations, promoting scalar mixing. However, this benefit is counterbalanced by a rise in frictional pressure drop per unit length, directly escalating pumping costs.

#### 3.1.2. Pressure Drop Characteristics of the Mixture

**Figure 14:**
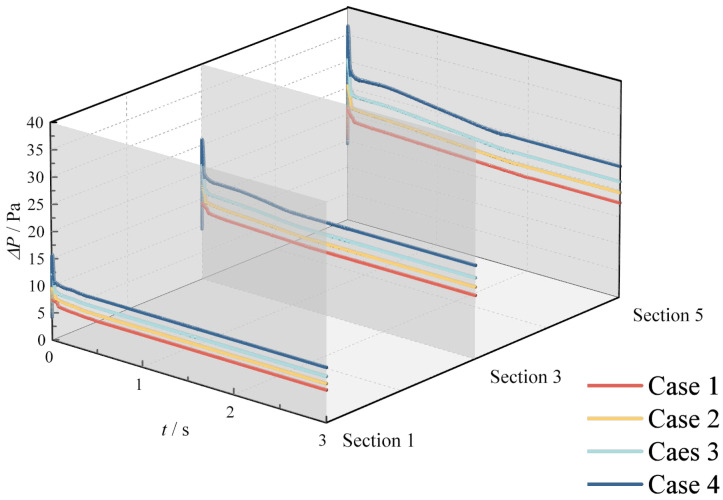
Temporal variation in pressure drop in the monitoring section with respect to changes in hydrogen mixing ratios (HMRs).

**Figure 15:**
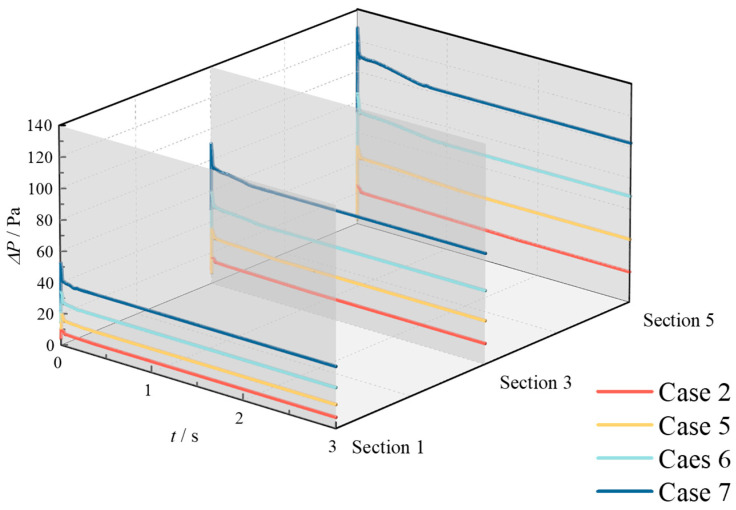
Temporal variation in pressure drop in the monitoring section with respect to changes in methane flow rate.

Figure 14 and Figure 15 depict the temporal variation in pressure drop across different monitoring sections under various operating conditions. The pressure drop, defined as the difference between the monitoring section and the methane inlet section, exhibits a consistent trend across conditions: an initial rapid increase, followed by a sharp decrease, and finally a gradual decline until stabilization. This trend can be attributed to the following mechanisms: (1) At the onset of hydrogen injection, the sudden increase in flow rate within the pipeline causes an immediate rise in pressure drop across all sections. (2) Initially, the T-junction hydrogen-blended natural gas pipeline is filled with methane. As hydrogen is introduced, methane in the hydrogen injection branch pipe is rapidly discharged. Given the significantly higher density of methane compared to hydrogen, the discharge process induces a rapid increase in pressure drop, followed by an immediate decrease. (3) Once methane is completely discharged from the branch pipe, the hydrogen content in the pipeline progressively increases, leading to a reduction in the density of the mixed gas. Consequently, the pressure drop decreases gradually. (4) Finally, as the hydrogen content stabilizes, the pressure drop reaches a steady state.

The key distinction between increasing HMR and increasing methane flow rate lies in their impact on the pressure drop. While both measures elevate the pressure drop across sections, increasing HMR results in only a marginal increase in flow rate. In contrast, increasing the methane flow rate significantly enhances the pipeline flow rate, thereby causing a more substantial rise in pressure drop.

**Figure 16:**
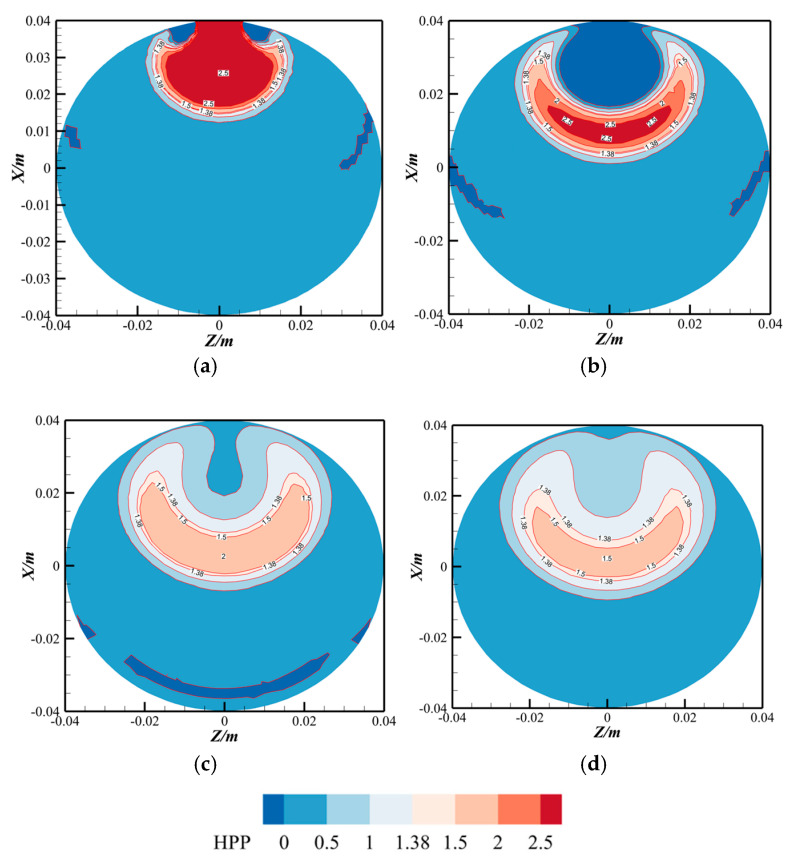
Partial pressure characteristics of hydrogen in Case 2 at 3 s. (**a**) *Y* = 5 mm; (**b**) *Y* = 30 mm; (**c**) *Y* = 60 mm; (**d**) *Y* = 90 mm.

Hydrogen partial pressure (HPP) represents a critical safety parameter in hydrogen-blended natural gas pipelines, distinct from pressure drop considerations that primarily govern transportation economics. Figure 16 illustrates the HPP contours at various *Y*-axis positions, calculated through a user-defined field function in ANSYS FLUENT (HPP = hydrogen molar fraction × total system pressure). As illustrated in the figure, the hydrogen partial pressure distribution exhibits distinct morphological patterns across different cross-sections. At the position 5 mm downstream of the hydrogen injection point (*Y* = 5 mm), the hydrogen partial pressure displays an inverted mushroom-shaped profile in the *XZ* plane. In contrast, at other axial locations, the distribution approximates a horseshoe-shaped pattern, with the highest partial pressure concentrated in the central region (≈2.5 MPa) and gradually decreasing radially outward (0.5–2.5 MPa).

According to ASME B31.12 specifications, material upgrades or protective coatings become mandatory when localized HPP exceeds 1.38 MPa. Based on this criterion, our analysis reveals that the 12 o’clock position of the pipeline at *Y* = 5 mm represents the most vulnerable location for hydrogen-induced safety risks. However, as the flow progresses downstream, the risk profile shifts: the 2 o’clock and 10 o’clock positions emerge as higher-risk zones, necessitating prioritized monitoring during pipeline operation.

In this case, the hydrogen partial pressure threshold (indicated by a flesh-pink contour) is visually discernible in the figure. Notably, no threshold exceedance is observed near the pipeline wall at locations beyond *Y* = 5 mm. This implies that hydrogen embrittlement risks are predominantly localized within a short downstream region near the injection point, with significantly lower risks along the remainder of the pipeline.

#### 3.1.3. Distribution Characteristics of the Hydrogen Mole Concentration

1. The influence of HMR and methane flow rate

To investigate the influence of HMR and methane flow rate on hydrogen mixing, the momentum ratio was introduced as a key parameter. The momentum ratio is defined as the ratio of the momentum of the hydrogen jet in the branch pipe to the momentum of the methane fluid in the main pipeline, expressed as follows [46]:

(16) $$M_{R}=\frac{\rho_{2}v_{2}^{2}}{\rho_{1}v_{1}^{2}}$$

where ρ_1_ represents the density of methane; *v*_1_ denotes flow rate of methane; ρ_2_ indicates the density of hydrogen; and *v*_2_ signifies the flow rate of hydrogen.

**Figure 17:**
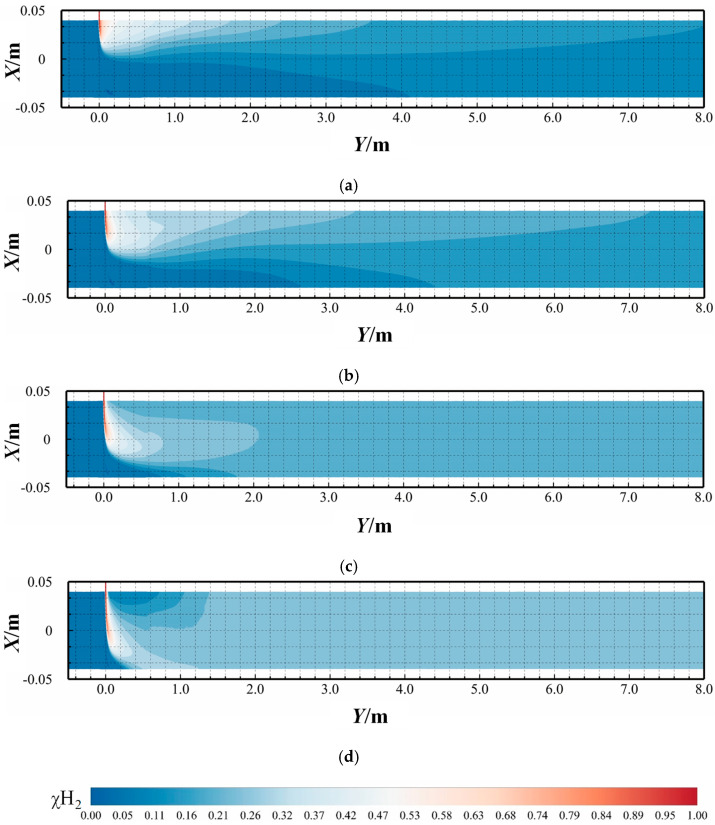
Distribution of mole concentration of hydrogen at different HMRs at 3 s. (**a**) *M*_R_ = 0.95 (HMR = 10%); (**b**) *M*_R_ = 2.39 (HMR = 15%); (**c**) *M*_R_ = 4.79 (HMR = 20%); (**d**) *M*_R_ = 8.51 (HMR = 25%).

Figure 17 illustrates the distribution of mole concentration of hydrogen (χH2) at various hydrogen mixing ratios (HMRs) at 3 s. As shown in Figure 17a–d, the HMR increases from 10% to 25%. In this study, the HMR was adjusted by increasing the hydrogen flow rate. As the HMR increases, the hydrogen flow rate rises, leading to an increase in the momentum ratio between the branch fluid and the main fluid. Consequently, the hydrogen jet penetrates the methane fluid more effectively. Specifically, the momentum ratio increases from 0.95 to 8.51 with the elevation of the HMR.

As shown in the figure, at lower momentum ratios, the jet injected from the branch pipe enters the main pipe, and its flow direction is immediately deflected due to the buoyancy difference between hydrogen and methane. This results in hydrogen being confined to the upper region of the pipe. In contrast, at higher hydrogen flow rates, the increased momentum allows the hydrogen jet to penetrate the methane fluid more efficiently, leading to hydrogen enrichment at the bottom of the main pipe. Notably, gravitational stratification develops progressively along the X-direction, while axial stratification manifests predominantly within the proximal region of the hydrogen injection point.

**Figure 18:**
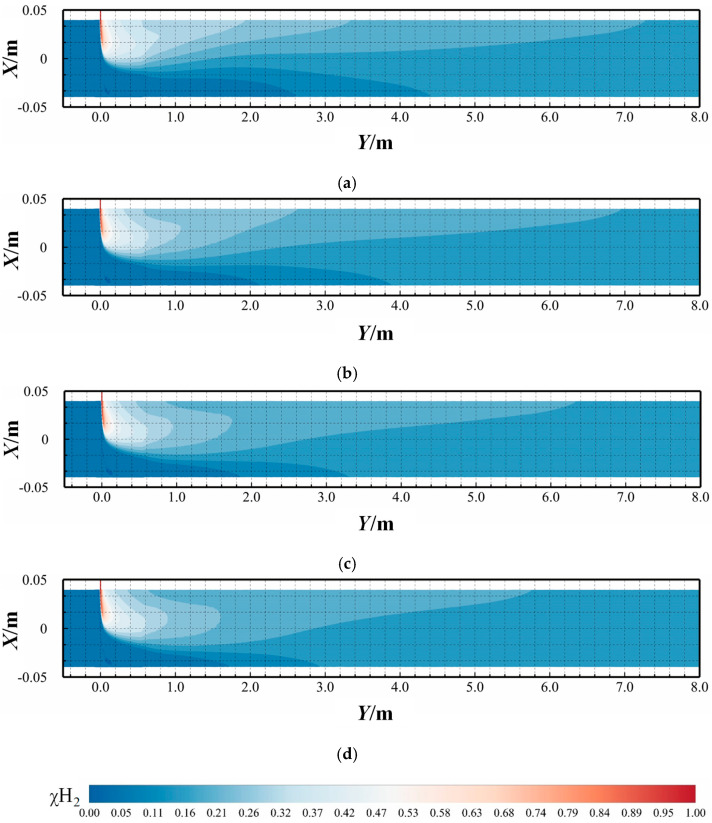
Distribution of mole concentration of hydrogen at different methane flow rates at 3 s. (**a**) *M*_R_ = 2.39 (*v*_1_ = 4 m/s); (**b**) *M*_R_ = 2.39 (*v*_1_ = 6 m/s); (**c**) *M*_R_ = 2.39 (*v*_1_ = 8 m/s); (**d**) *M*_R_ = 2.39 (*v*_1_ = 10 m/s).

Figure 18 shows the distribution of hydrogen mole concentration (χH2) at different methane flow rates at 3 s. In Figure 18a–d, the methane flow rate increases from 4 m/s to 10 m/s. As the methane flow rate increases, the HMR remains constant and both the methane and hydrogen flow rates increase proportionally, resulting in an unchanged momentum ratio. Consequently, the depth of hydrogen jet penetration into the main pipeline and the position of deflection remains consistent.

In conclusion, the gas stratification phenomenon in the main pipeline can be categorized into gravitational stratification and axial stratification (along the length direction of the pipeline). Gravitational stratification is primarily driven by the density disparity between methane and hydrogen, where methane exhibits higher density compared to hydrogen. Axial stratification arises from two key factors: when the hydrogen jet-to-methane flow rate ratio exceeds critical thresholds and the hydrogen jet penetration depth increases significantly. Therefore, altering the HMR primarily modifies the buoyancy effect, thereby influencing the radial mixing process of hydrogen–methane in the pipeline. These conditions induce vortex dynamics in the injection zone, causing hydrogen migration from lateral regions to the upper section of the pipeline, thereby creating axial concentration gradients.

2. The phenomenon of radial stratification

**Figure 19:**
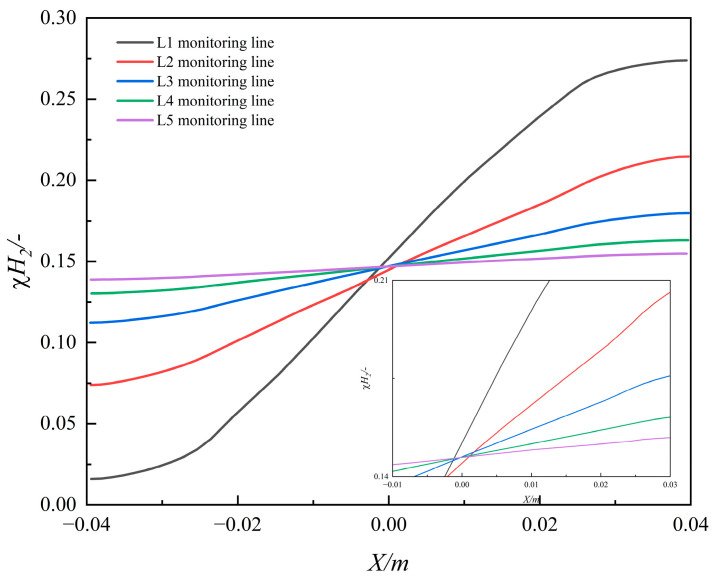
Radial distribution of hydrogen mole fraction in Case 2 at 3 s.

To elucidate the radial stratification of hydrogen–methane mixing dynamics, Figure 19 presents the hydrogen molar fraction profiles for Case 2 at 3 s. Monitoring lines L1–L5 were positioned at axial distances of Y = 20 D, 40 D, 60 D, 80 D, and 100 D along the pipeline, spanning from the pipe bottom (X = −0.04 m) to the top (X = 0.04 m) through the cross-sectional centerline.

The data reveal pronounced hydrogen stratification under steady flow conditions. At a fixed axial position (e.g., L1), the hydrogen molar fraction at the pipe bottom (≈0.03) is significantly lower than that at the top (≈0.7). A mild concentration gradient (≈2.5 m⁻^1^) is observed within the near-wall region (0.1 D from the wall), whereas a steeper, uniform gradient (≈5.6 m⁻^1^) dominates the central flow domain. This stratification arises from the buoyancy-driven accumulation of low-density hydrogen at the pipe crown, coupled with limited penetration depth due to the low momentum of transverse hydrogen injection. Such persistent crown enrichment poses operational risks, as prolonged exposure to elevated hydrogen concentrations accelerates permeation-driven hydrogen embrittlement in pipeline materials.

Axial progression (L1→L5) demonstrates progressive homogenization, with the hydrogen molar fraction converging to 0.15 across the entire cross-section at Y = 100 D. This homogenization is attributed to turbulent diffusion, where vortical structures progressively stretch and entangle hydrogen–methane interfaces, enhancing scalar mixing efficiency with increasing residence time. The diminishing radial gradient (∂C/∂X decreasing from 5.6 m⁻^1^ to less than 0.1 m⁻^1^) quantitatively confirms the dominance of turbulent dispersion over buoyancy effects at downstream locations.

3. Distribution of mole concentration variance of hydrogen

To deepen the hydrodynamic understanding of methane–hydrogen mixing dynamics, the hydrogen mole fraction variance (HMFV) is introduced as a quantitative metric to assess mixing heterogeneity, where higher variance values indicate greater spatial nonuniformity in hydrogen distribution. The HMFV is mathematically defined as

(17) $$\sigma^{2}=\frac{1}{N}\sum_{i=1}^{N}{\left(y_{H_{2},i}-\bar{y_{H_{2}}}\right)}^{2}$$

where $y_{H_{2},i}$ represents the hydrogen mole fraction at grid; $\bar{y_{H_{2}}}$ is the domain-averaged hydrogen mole fraction; and N denotes the total number of computational grids.

**Figure 20:**
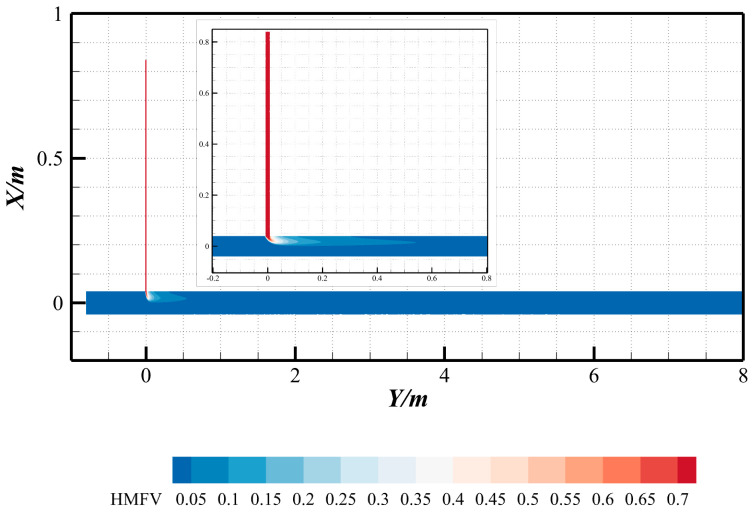
Distribution of mole concentration variance of hydrogen in Case 2 at 3 s.

Using the custom field function capability in ANSYS Fluent, the spatial distribution of HMFV was computed (Figure 20). The results reveal that, except for regions near the hydrogen injection port where significant mole fraction disparities between the injector and main pipeline yield anomalously high variance values, the HMFV distribution remains relatively uniform across the pipeline. A pronounced variance gradient is observed only within a short downstream distance (~7.5 D) post-injection. Hydrogen injection generates a dominant transverse shear layer, characterized by steep gradients in both velocity and hydrogen mole fraction variance. These gradients trigger Kelvin–Helmholtz instabilities, leading to the formation of turbulent vortex structures. These vortices amplify interfacial contact between methane and hydrogen through repetitive stretching and folding mechanisms, thereby enhancing turbulent mixing.

**Figure 21:**
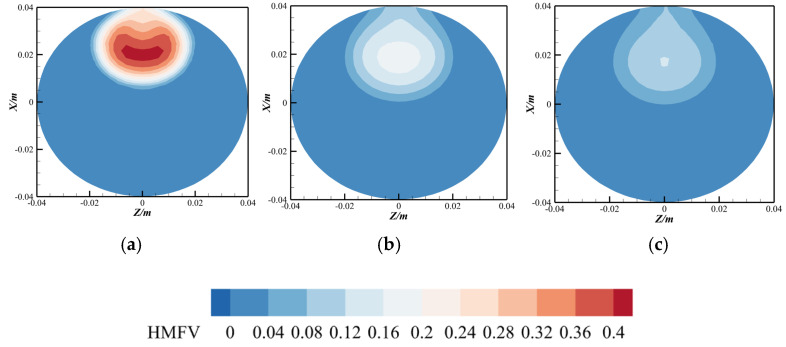
Distribution of mole concentration of hydrogen in Case 2 at 3 s. (**a**) *Y* = 30 mm; (**b**) *Y* = 60 mm; (**c**) *Y* = 90 mm.

Figure 21 illustrates the spatial distribution of hydrogen mole fraction variance across various axial positions (*Y*-axis). The results reveal that the variance is predominantly concentrated near the top region of the pipeline, indicating significant fluctuations in hydrogen mole fraction and pronounced mixing heterogeneity, which suggests the persistence of radial stratification. As the flow progresses downstream (i.e., with increasing *Y*-values), the spatial disparity in hydrogen mole fraction variance diminishes progressively. This trend demonstrates that radial stratification—initially driven by buoyancy effects—is gradually attenuated by turbulent dispersion, leading to enhanced homogenization of the hydrogen–methane mixture. The observed homogenization aligns with the intensified turbulent momentum transport and the progressive breakdown of density-driven layering at larger axial distances.

4. The effect of hydrogen injection angle

**Figure 22:**
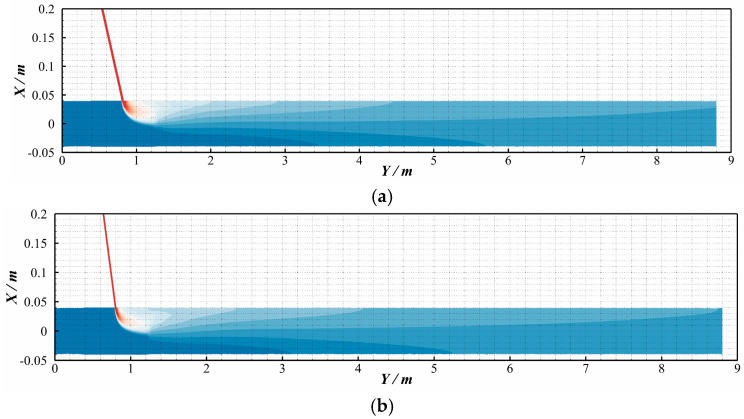

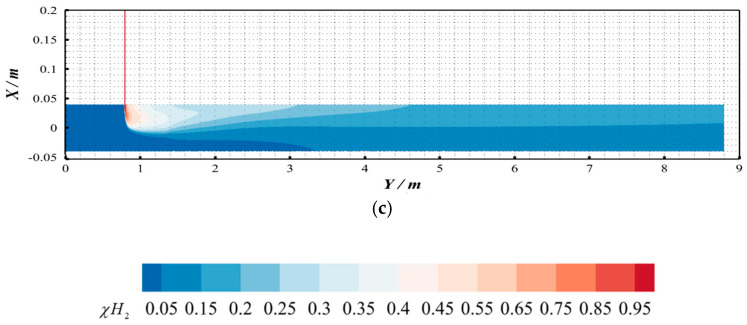
Distribution of hydrogen mole concentration under varying injection angles. (**a**) 30°; (**b**) 45°; (**c**) 90°.

In addition to the hydrogen-to-methane ratio (HMR) and methane flow velocity, the hydrogen injection angle significantly influences the mixing efficiency. Figure 22 presents hydrogen molar fraction contours under different injection angles: (a) 30°, (b) 45°, and (c) 90°. The results demonstrate that, under identical flow conditions, the impact of the injection angle on hydrogen distribution is primarily confined within 25 D downstream. A smaller injection angle generally correlates with a more prolonged mixing process over longer axial distances. As the injection angle increases, the radial penetration depth of hydrogen improves, leading to a gradual attenuation of radial stratification. At a 90° injection, a pronounced transverse shear layer forms, characterized by steep velocity gradients that trigger Kelvin–Helmholtz instabilities. These instabilities generate turbulent vortex structures, which enhance hydrogen–methane interfacial contact through vortex stretching and folding, thereby accelerating turbulent mixing. However, this configuration also induces higher energy dissipation, resulting in faster decay of mixing efficiency downstream. In contrast, oblique injection angles (30° or 45°) produce inclined shear layers, promoting the formation of streamwise vortex pairs. These vortices distribute turbulent kinetic energy more uniformly across the flow field, sustaining the mixing process over extended distances.

**Figure 23:**
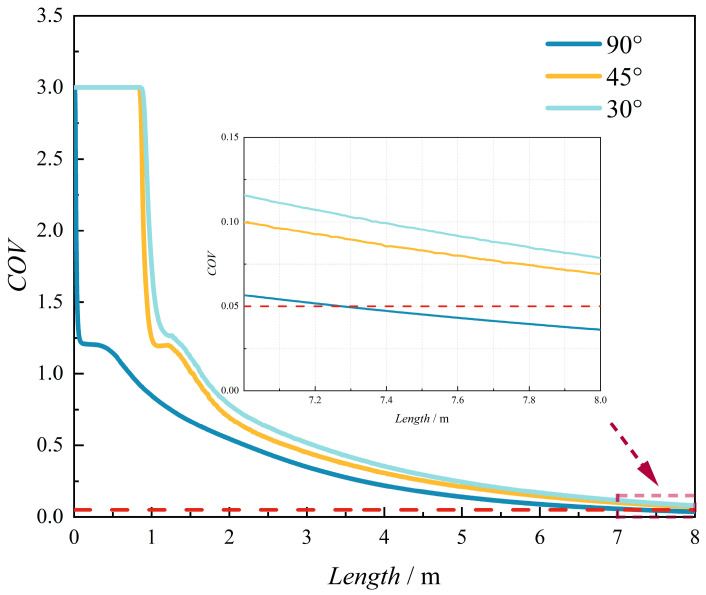
Distribution of COV under varying injection angles.

Figure 23 illustrates the axial evolution of the coefficient of variation (COV) under different hydrogen injection angles, with the red dashed line denoting the homogeneity threshold (COV ≤ 0.05). The results demonstrate that for 90° injection, the COV decreases rapidly within a short axial distance (L < 5 D) and subsequently approaches a steady-state value with further downstream progression. In contrast, at 30° and 45° injection angles, the COV remains nearly constant over an initial 12.5 D pipeline segment before gradually declining to equilibrium. Comparatively, the 90° configuration achieves homogeneity over a shorter pipeline length. This behavior is attributed to the prominent transverse shear layer formed under perpendicular injection, which induces strong velocity gradients and triggers Kelvin–Helmholtz instabilities. These instabilities enhance turbulent mixing through vortex stretching and folding mechanisms, accelerating initial homogenization. However, the intensified energy dissipation associated with this turbulent cascade leads to faster decay of mixing efficiency downstream, limiting sustained homogenization. Conversely, oblique injection angles (30–45°) generate inclined shear layers that promote streamwise vortices, distributing turbulent kinetic energy more uniformly and prolonging mixing effectiveness over extended distances.

#### 3.1.4. Temporal Evolution of the Hydrogen Mole Concentration

**Figure 24:**
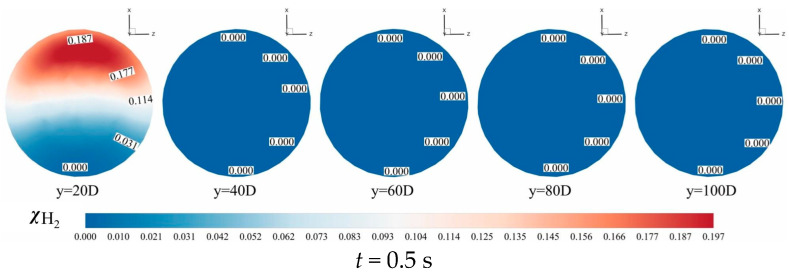

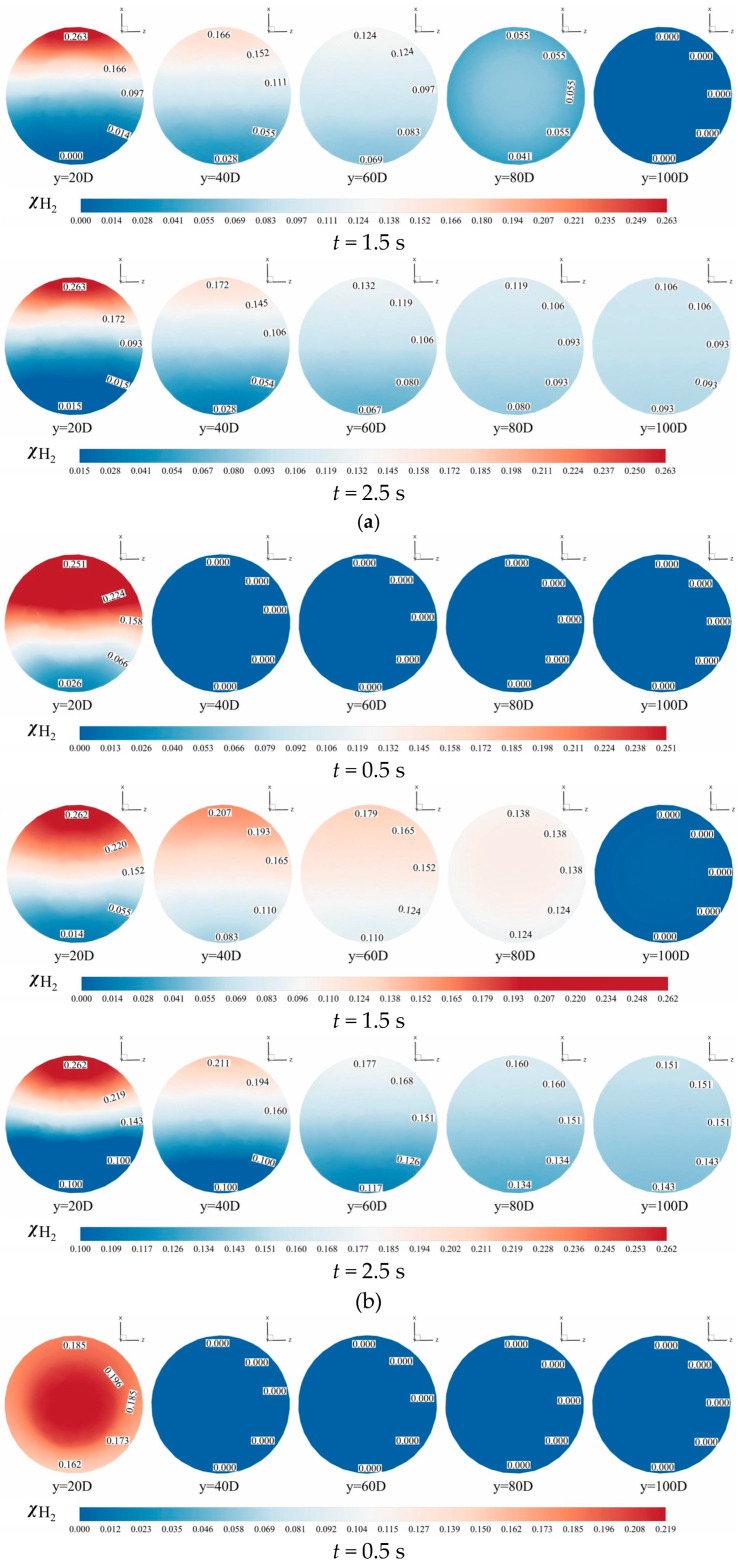

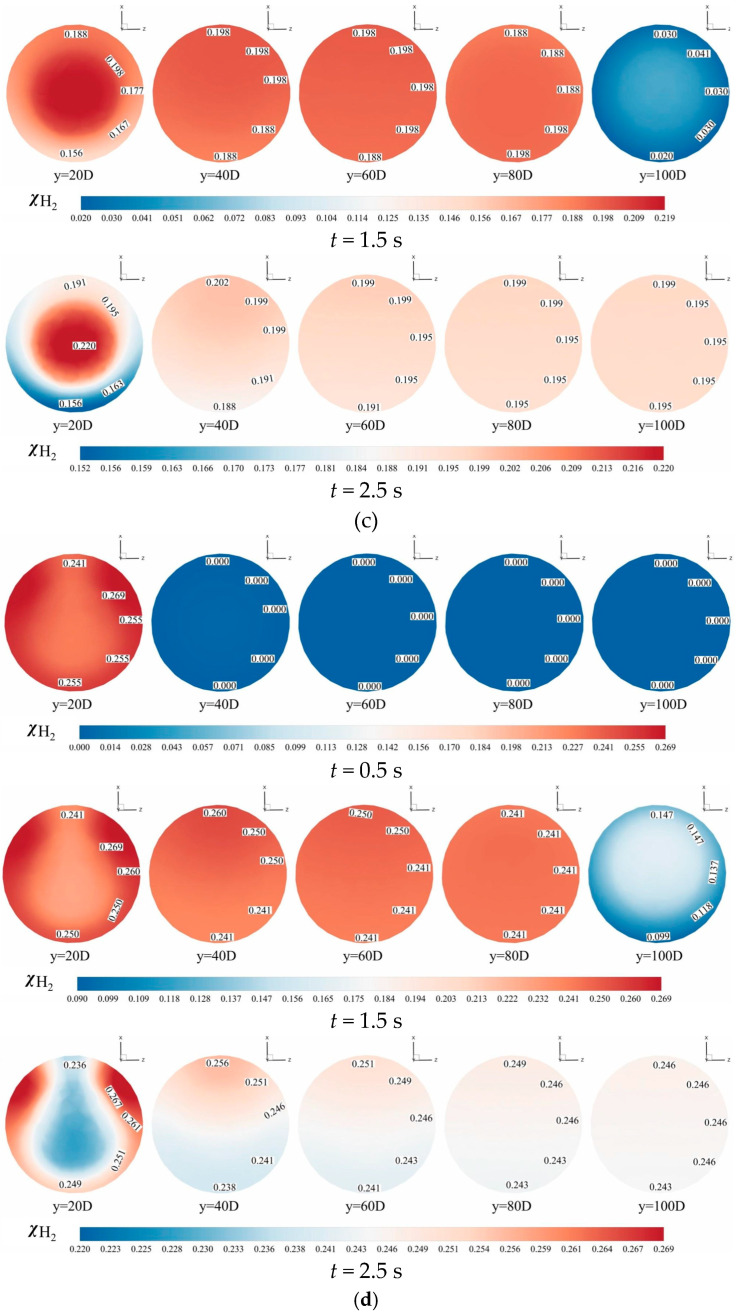
Spatial and temporal distribution of hydrogen mole concentration under varying hydrogen mixing rates (HMRs). (**a**) HMR = 10%; (**b**) HMR = 15%; (**c**) HMR = 20%; (**d**) HMR = 25%.

**Figure 25:**
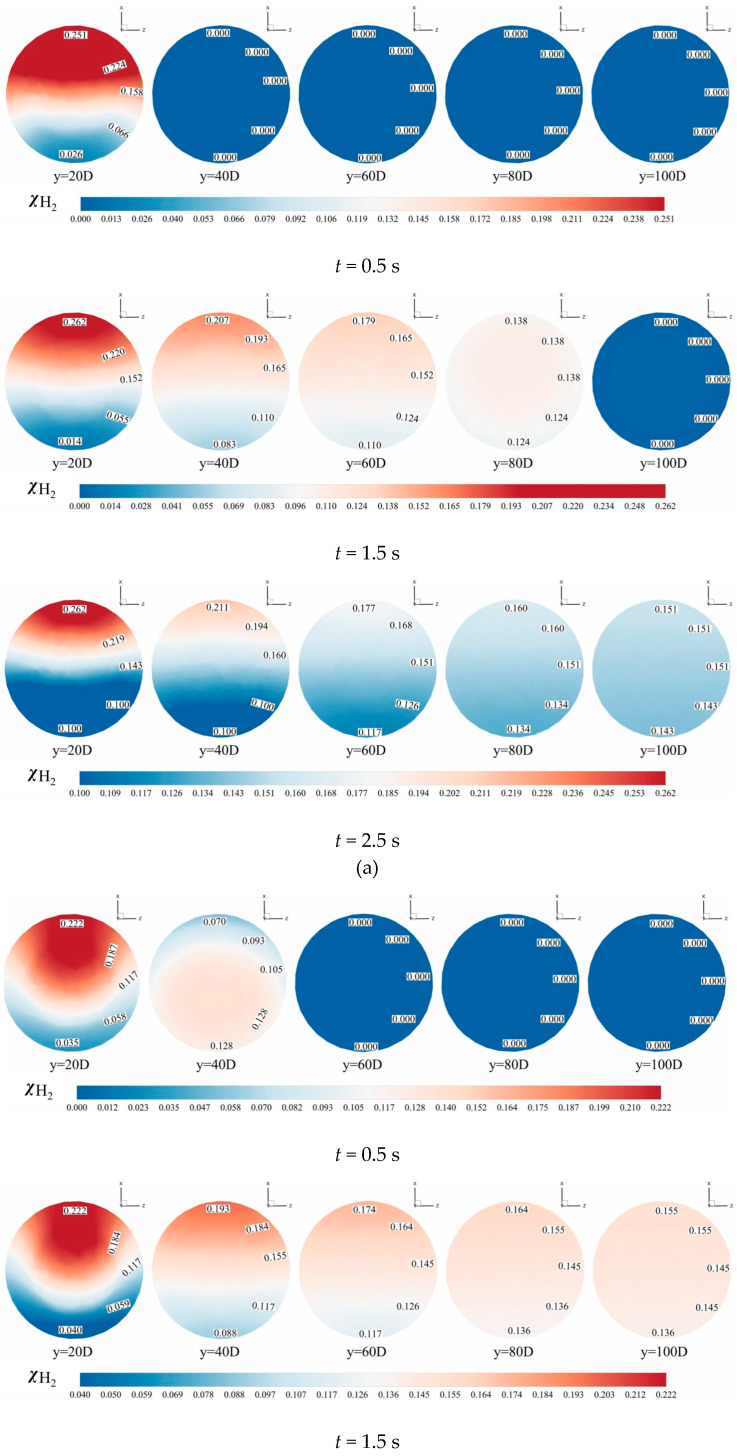

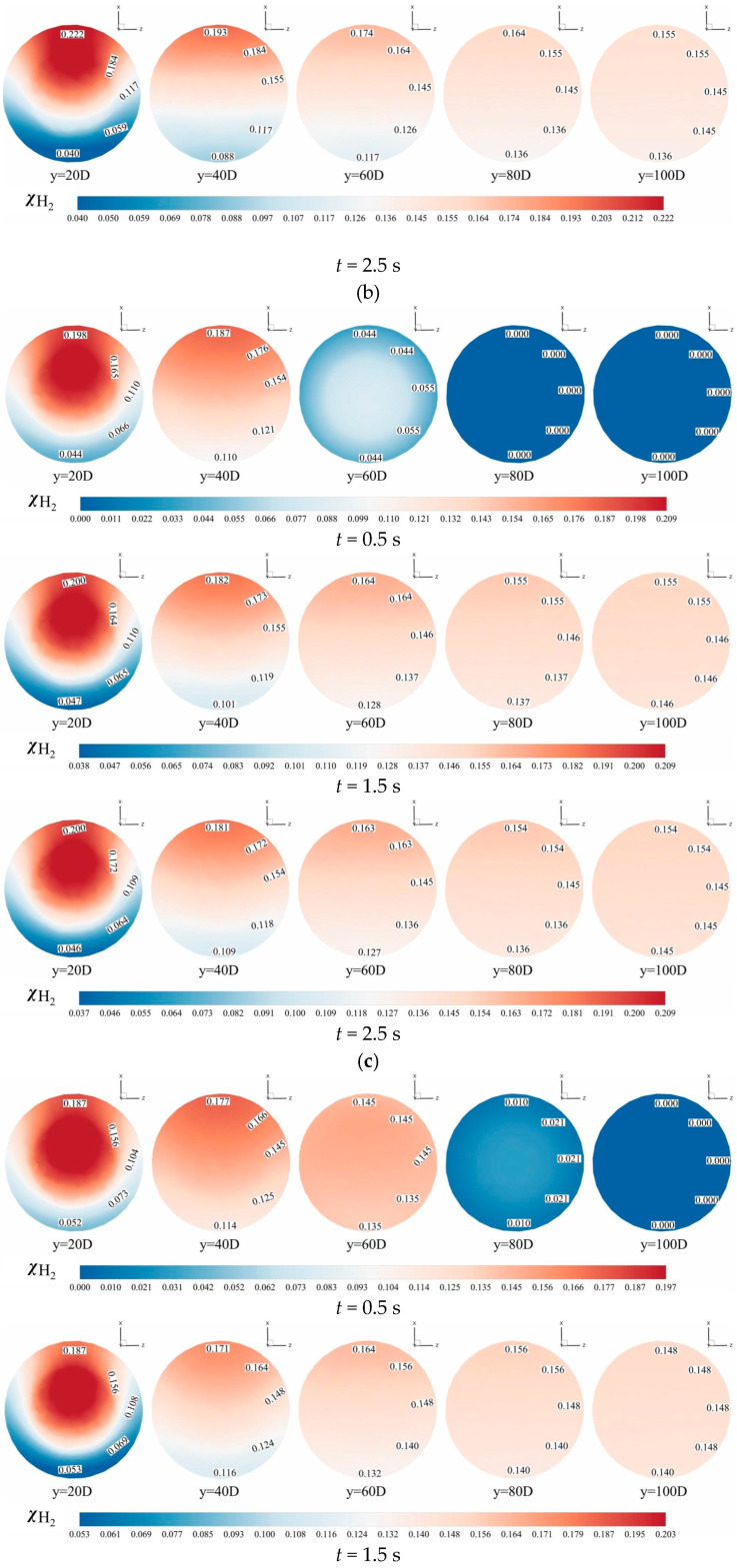

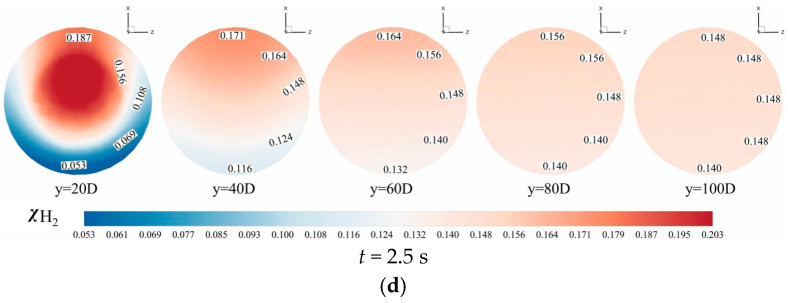
Spatial and temporal distribution of hydrogen mole concentration under varying methane flow rates. (**a**) *v*_1_ = 4 m/s; (**b**) *v*_1_ = 6 m/s; (**c**) *v*_1_ = 8 m/s; (**d**) *v*_1_ = 10 m/s.

Figure 24 and Figure 25 present contours of hydrogen mole concentration distribution (χH2) for the seven experimental groups at *t* = 0.5 s, *t* = 1.5 s, and *t* = 2.5 s. By comparing the hydrogen mole concentration distribution at identical positions under different operating conditions and time, it is evident that the distribution becomes increasingly uniform over time at locations farther from the hydrogen mixing point. However, closer to the mixing point, hydrogen stratification remains pronounced and persists over time. This phenomenon arises because the flow rate of hydrogen near the mixing point remains consistently higher than at other positions, hindering effective mixing with methane, thus impeding the mixing process.

From the two contours, it is apparent that hydrogen and methane stratification primarily occurs along the gravitational axis within the pipeline. This stratification is fundamentally attributed to the density difference between hydrogen and methane, where hydrogen, being less dense, tends to accumulate at the top of the pipeline, while methane, being denser, concentrates at the bottom under the influence of gravity. However, as flow time progresses and mixing length increases, this gravitational stratification gradually diminishes. At 2.5 s, in the outlet, the difference in hydrogen mole concentration between the top and bottom of the cross-section does not exceed 0.02, under various operating conditions, indicating a significant improvement in stratification.

From Figure 24, it is evident that hydrogen stratification occurs not only along the gravitational axis but also in the radial direction as the hydrogen injection amount increases. Specifically, the hydrogen mole concentration distribution at the cross-section *Y* = 20 *D* in Figure 24d clearly demonstrates radial stratification within the pipeline. As the hydrogen mixing rate (HMR) increases, the phenomenon of radial stratification is more pronounced. Concurrently, the depth of hydrogen injection increases with this velocity ratio. Unlike scenarios with low hydrogen injection amounts, the direction of hydrogen movement shifts, requiring hydrogen to ascend from the bottom to the top of the pipe. However, due to the higher flow velocity at the center of the pipe compared to the lower velocity near the pipe walls, hydrogen moves upward along the low-velocity regions on both sides of the pipeline. This results in a configuration where hydrogen near the pipe walls envelops the methane flow at the center of the pipeline.

**Figure 26:**
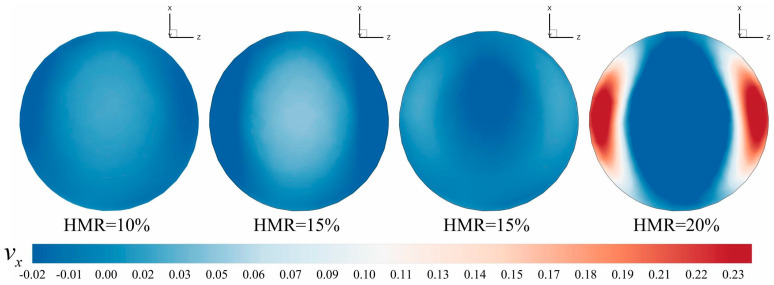
Velocity contour in *X*-direction on the monitoring surface at *Y*= 20 *D*, captured at *t* = 2.5 s with different hydrogen mixing ratios (HMRs). Note: The symbol *v_x_* represents the velocity component in the X-direction.

To substantiate this observation, Figure 26 presents the *X*-direction velocity contour of the *Y* = 20 *D* monitoring surface at 2.5 s under varying hydrogen mixing ratios (HMRs). As the hydrogen mixing ratio (HMR) increases, the *X*-direction velocity transitions from negative to positive near the inner walls of the pipeline, while the velocity at the center of the pipeline remains positive. This indicates a reversal in the direction of hydrogen movement from downward to upward, a phenomenon that persists over time. However, unlike gravitational stratification, radial stratification is confined to regions proximate to the hydrogen mixing point and diminishes with increasing distance. In contrast, gravitational stratification typically extends over greater distances compared to radial stratification.

### 3.2. Mixing Behaviors of Hydrogen–Methane Mixtures

To comprehensively investigate the gas mixing behaviors of hydrogen–methane blending, two quantitative evaluation metrics were established: the uniform mixing length (defined as the minimum axial distance required for the first pipeline cross-section to achieve 95% mixing uniformity, which corresponds to a coefficient of variation (COV) value less than or equal to 5% (COV ≤ 0.05)) and the initial uniform mixing time (defined as the duration required for the first pipeline cross-section to achieve 95% mixing uniformity, which corresponds to a coefficient of variation (COV) value less than or equal to 5% (COV ≤ 0.05)). These parameters were specifically developed to analyze the mixing uniformity from both spatial and temporal perspectives, aiming to optimize hydrogen injection strategies and ensure rapid and homogeneous mixing of the hydrogen–methane.

#### 3.2.1. Uniform Mixing Length

**Figure 27:**
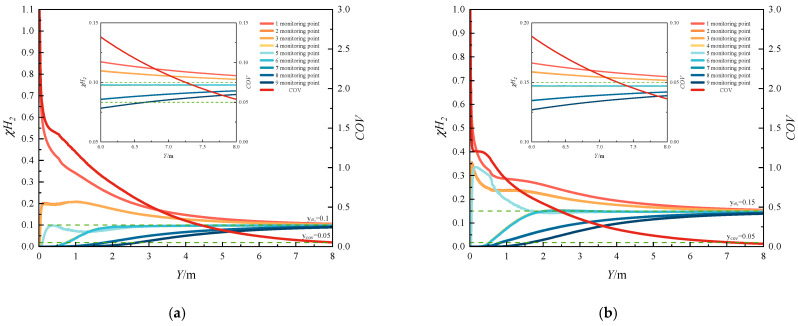

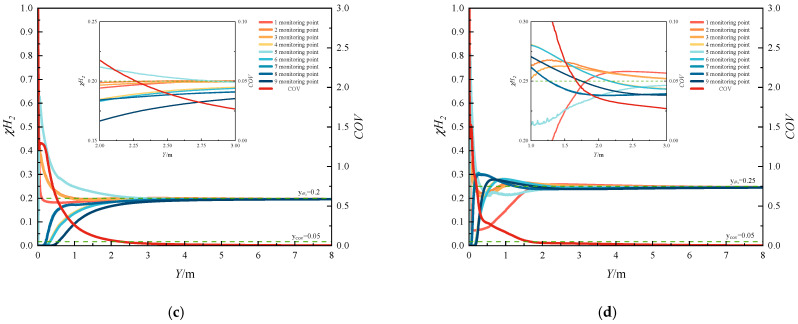
Spatial distribution of hydrogen mole concentration and the COV along the pipeline for different HMRs (**a**) HMR = 10%; (**b**) HMR = 15%; (**c**) HMR = 20%; (**d**) HMR = 25%.

**Figure 28:**
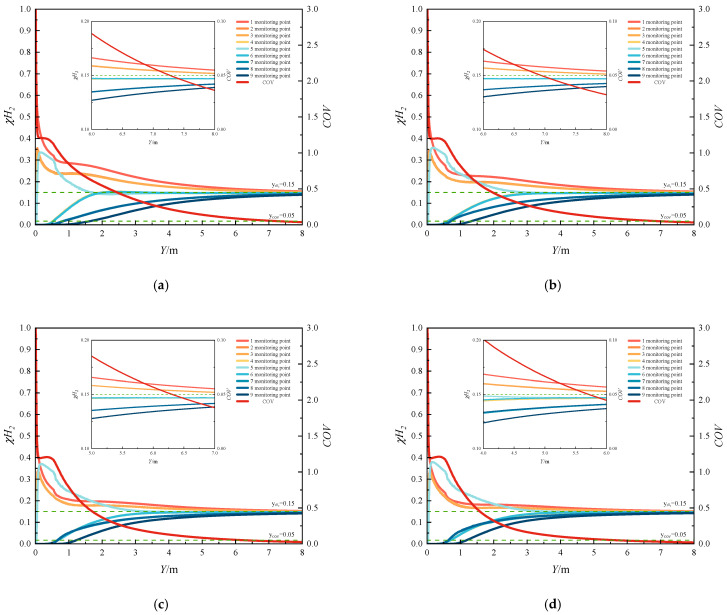
Spatial distribution of hydrogen mole concentration and the COV along the pipeline for different methane flow rates. (**a**) *v*_1_ = 4 m/s; (**b**) *v*_1_ = 6 m/s; (**c**) *v*_1_ = 8 m/s; (**d**) *v*_1_ = 10 m/s.

Figure 27 and Figure 28 present the spatial variations in the COV and hydrogen mole concentration along the pipeline under different operating conditions. The results demonstrate that the mole fraction of hydrogen at all monitoring points converges to a consistent value with increasing distance. The COV profile exhibits a characteristic three-stage pattern: (1) an initial rapid decline, (2) a subsequent phase of minimal reduction or slight increase, and (3) a final steady decrease. This observed trend can be attributed to the hydrodynamic behavior of the gas mixture. Initially, the vertical injection of the hydrogen jet at the mixing point creates a significant concentration gradient, resulting in a sharp decrease in COV. As the jet propagates downstream, the methane flow gradually alters the jet orientation from vertical to horizontal alignment. During this transitional phase, the velocity discrepancy between the hydrogen jet and surrounding methane hinders effective mixing, causing the COV to exhibit minimal reduction or even a slight increase. Eventually, as the hydrogen velocity equilibrates with the methane flow velocity, the mixing efficiency improves significantly. The uniformity reduction rate experiences a secondary slowdown when the COV decreases below 0.05, indicating the approach to complete mixing.

Figure 27 illustrates the influence of HMR on the COV and hydrogen mole concentration distribution. The results demonstrate that with increasing HMRs, the COV profile exhibits a more pronounced secondary increase following its initial rapid decline downstream of the injection point. This phenomenon can be attributed to the enhanced jet velocity associated with higher HMRs, which creates a substantial velocity gradient that impedes methane entrainment near the injection region, consequently leading to an increase in the COV rather than the expected decrease.

Furthermore, increased HMRs result in greater jet penetration depth, significantly affecting the spatial distribution of hydrogen mole concentration. Specifically, the hydrogen mole concentration at the pipeline bottom exhibits a rapid increase at locations closer to the mixing point, with the rate of increase being positively correlated with HMR. This observation indicates that higher injection rates accelerate the establishment of hydrogen concentration gradients along the vertical axis of the pipeline.

Figure 28 demonstrates the impact of methane flow rate variations on the COV and mole fraction distribution. Under constant HMR conditions, where the ratio of hydrogen jet flow rate to methane flow rate remains unchanged, the injection depth and spatial distribution of hydrogen mole concentration exhibit minimal variations. However, increased methane flow rates significantly accelerate the reduction in the COV, leading to shorter mixing times and enhanced hydrogen–methane mixing efficiency.

**Figure 29:**
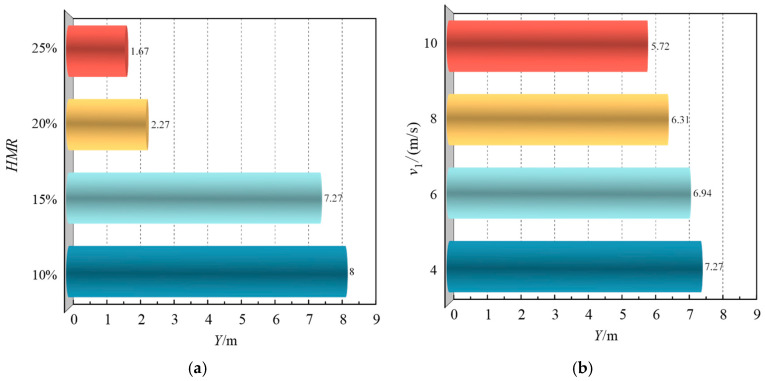
Comparison of gas uniform mixing length under different operating conditions. (**a**) Variation in HMR; (**b**) Variation in methane flow rate.

Figure 29 presents the uniform mixing length for achieving a COV below 0.05 under seven operational conditions. Figure 29a demonstrates the influence of HMR on uniform mixing length. As the HMR increases from 10% to 25%, the uniform mixing length decreases significantly from 100 *D* to 20.875 *D*. This reduction is primarily governed by the ratio of hydrogen jet flow rate to methane flow rate, which follows an ascending order of 2.88, 4.42, 6.27, and 8.43 for groups 1 to 4, respectively. Notably, when this ratio increases from 4.42 to 6.27, a substantial reduction in uniform mixing length is observed, suggesting that optimal hydrogen injection rates can enhance hydrogen–methane mixing efficiency.

Figure 29b illustrates the effect of methane flow rate on uniform mixing length. Increasing the flow velocity from 4 m/s to 10 m/s reduces the uniform mixing length from 90.875 *D* to 71.5 *D*. This phenomenon can be attributed to the increased Reynolds number in the main pipeline, which enhances turbulence intensity, thereby promoting more efficient gas mixing.

It is crucial to note that the HMR and velocity ratio are interdependent parameters in this study, as well as in most numerical simulations. As reported by Zhang et al. [20], increasing the HMR (corresponding to an increase in the hydrogen jet flow rate to methane flow rate ratio from 0.05 to 0.2) can increase the uniform mixing length. This observation indicates that in T-junction hydrogen-blended natural gas pipelines, the relationship between HMR and uniform mixing length is not linear but is significantly influenced by the ratio of hydrogen jet flow rate to methane flow rate. Therefore, achieving uniform hydrogen–methane mixing requires careful optimization of both parameters rather than simply increasing the hydrogen injection rate.

#### 3.2.2. Initial Uniform Mixing Time

**Figure 30:**
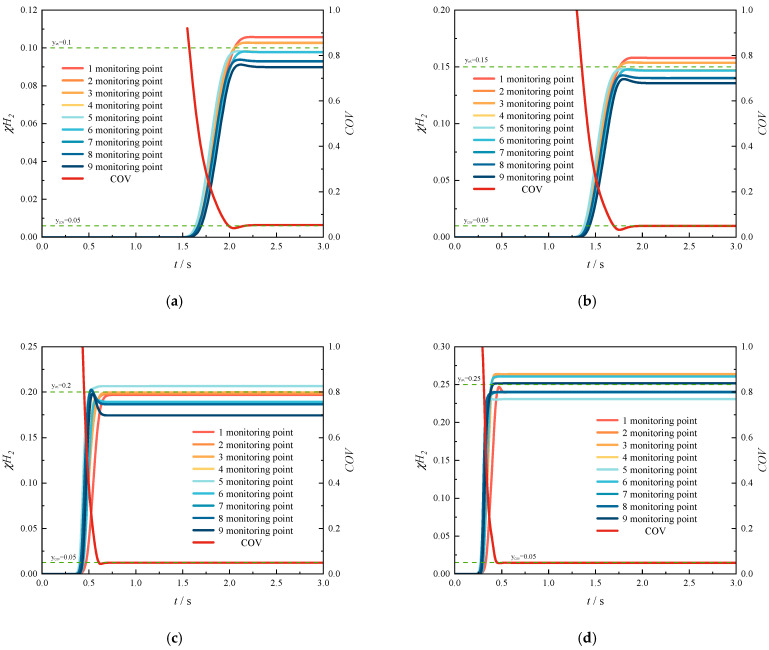
Hydrogen mole concentration and the COV over time for different HMRs. (**a**) HMR = 10%, *Y* = 100.000 *D*; (**b**) HMR = 15%, *Y* = 90.875 *D*; (**c**) HMR = 20%, *Y* = 28.375 *D*; (**d**) HMR = 25%, *Y* = 20.875 *D*.

**Figure 31:**
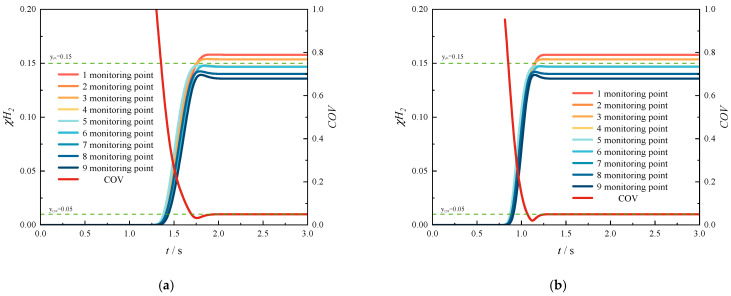

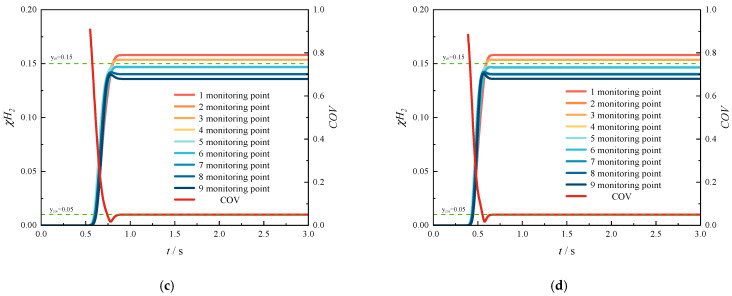
Hydrogen mole concentration and the COV over time at different methane flow rates. (**a**) *v*_1_ = 4 m/s, *Y* = 90.875 *D*; (**b**) *v*_1_ = 6 m/s, *Y* = 86.750 *D*; (**c**) *v*_1_ = 8 m/s, *Y* = 78.875 *D*; (**d**) *v*_1_ = 10 m/s, *Y* = 71.500 *D*.

Figure 30 and Figure 31 illustrate the temporal variations in the COV and hydrogen mole concentration under different operating conditions. The initial uniform mixing time is defined as the time required for the first cross-section in the pipeline to achieve uniform mixing of hydrogen and methane. Consequently, as illustrated in Figure 17 and Figure 18, the investigated cross-sectional positions vary under different operating conditions. As shown in the figures, the hydrogen mole concentration at monitoring point 5 increases first, because this point is located at the center of the pipeline, where the highest-velocity hydrogen arrives earliest. Subsequently, the hydrogen mole concentration at other monitoring points begins to rise. The temporal evolution of COV in each section exhibits an initial rapid decrease, followed by a slight increase, and finally stabilizes. Furthermore, refer to Figure S1 to observe temporal evolution of the hydrogen mole concentration at different monitoring cross-sections when changing HMR. It can be seen that the process from the initial flow of hydrogen through the monitoring section to the stabilization of hydrogen mole fraction at that section is extremely rapid. However, the uniformity of the mixture of hydrogen and methane cannot be guaranteed in a short period of time. This is consistent with the results of dispersion experiments on 10–30% hydrogen mixtures under static conditions conducted by Marangon et al. (2014) [48], which showed rapid initial mixing via turbulence followed by slower diffusion-driven homogenization. This behavior can be attributed to the following mechanism: hydrogen initially flows through the center of the section, while the remaining areas contain negligible hydrogen, resulting in a high COV. As hydrogen gradually diffuses to other regions of the section, the COV decreases rapidly. This reduction occurs because hydrogen first traverses the center of the section, leaving the surrounding areas with minimal hydrogen concentration, which results in an initially elevated COV. During this phase, the hydrogen mole concentration in the section has not yet reached the target value, and minor fluctuations arise due to incomplete mixing. Consequently, the stratification phenomenon remains insignificant. However, the gas uniformity becomes nearly ideal at this stage, with the COV potentially decreasing to values below 0.05. Eventually, the hydrogen mole concentration stabilizes at the target level, causing the COV to increase slightly and stabilize at a value close to but not exceeding 0.05.

Figure 30 illustrates the temporal variation in the COV under different HMRs. As shown in the figure, the COV exhibits a slight increase following an initial rapid decrease, and the magnitude of this increase diminishes with higher HMRs. This behavior can be attributed to the elevated hydrogen concentration within the section, resulting from an increased HMR. Although a small amount of hydrogen may not fully disperse, the significant density difference between hydrogen and methane within the section promotes stratification. Consequently, when the HMR is low, fluctuations in the COV are minimal. Furthermore, as the HMR increases, the initial uniform mixing length in the T-junction hydrogen-blended natural gas pipeline decreases progressively. Specifically, the initial uniform mixing time decreases from 2.41 s at an HMR of 10% to 0.46 s at an HMR of 25%. Notably, an initial uniform mixing time of 1.69 s is observed at an HMR of 15%, whereas only 0.61 s is required at an HMR of 20%. In other words, as the ratio of hydrogen jet flow rate to methane flow rate increases from 4.42 to 6.27, the initial uniform mixing length in the pipeline is significantly shortened, leading to a substantial reduction in initial uniform mixing time, and the mixing performance of hydrogen–methane is enhanced. For more detailed data, please refer to the attached Figure S2 Bar chart of uniform mixing time of hydrogen mole fraction at different monitoring sections when changing the HMR. From the figure, we can see the time required for the mole fraction of hydrogen to stabilize at each monitoring section from the start of hydrogen injection. This is consistent with the research results on the explosion behavior of natural gas and hydrogen mixtures conducted by Zheng in 2024 [49]. Increasing the volume fraction of hydrogen and the diffusion time of the hydrogen–methane mixture can improve the homogeneity of the hydrogen–methane–air mixture.

Figure 31 illustrates the temporal variation in the COV under different methane flow rates. A comparison reveals that increasing the methane flow rate significantly reduces the initial uniform mixing time. Specifically, as the methane flow rate increases from 4 m/s to 10 m/s, the initial uniform mixing time decreases from 1.69 s to 0.56 s. Overall, compared to the reduction in mixing time achieved by increasing the HMR, the decrease in mixing time is more consistent and evenly distributed with the increase in methane flow rate. Furthermore, there is no significant interval of abrupt reduction in mixing time observed.

By comparison, it can be found that increasing the flow rate can shorten the initial uniform mixing time in the pipeline. The methane flow rate increases from 4 m/s to 10 m/s, and the initial uniform mixing time is shortened from 1.69 s to 0.56 s. As a whole, compared with the time used to shorten the initial uniform mixing time by increasing the amount of hydrogen injection, the time is shortened more evenly with the increase in methane flow rate. There will not be any drastic changes in mixing time.

#### 3.2.3. Overall Uniform Mixing Time

**Figure 32:**
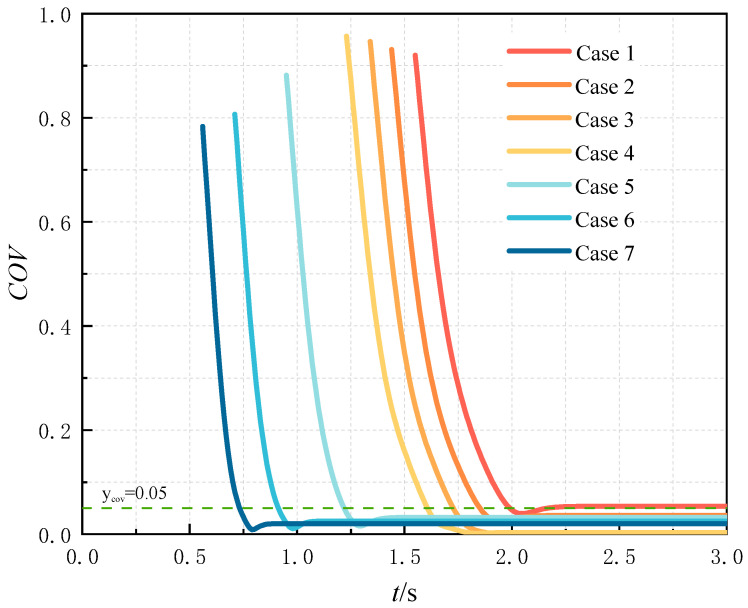
Temporal variation in the COV at the outlet section of the pipe.

To comprehensively assess the hydrogen–methane mixing efficiency, this study introduces the concept of overall mixing time, defined as the time required from the completion of uniform mixing at the first cross-section of the pipeline to the completion of uniform mixing at the outlet cross-section. It is mathematically expressed as the difference between the uniform mixing time at the outlet and the initial uniform mixing time. A systematic investigation was first conducted on the temporal evolution of the coefficient of variation (COV) at the pipe outlet cross-section, with the corresponding results presented in Figure 32. In Figure 32, cases 1 to 4 represent different HRM conditions ranging from 5% to 20%, while cases 2, 5, 6, and 7 correspond to distinct methane flow rate variations from 2 m/s to 8 m/s. The figure demonstrates that both increasing the HMR and adjusting the methane flow rate can reduce the outlet uniform mixing time (defined as the time required for hydrogen and methane to achieve uniform mixing at the pipeline outlet). Moreover, the temporal trend of mixing uniformity in the outlet section is consistent with that observed in the uniform mixing length.

**Figure 33:**
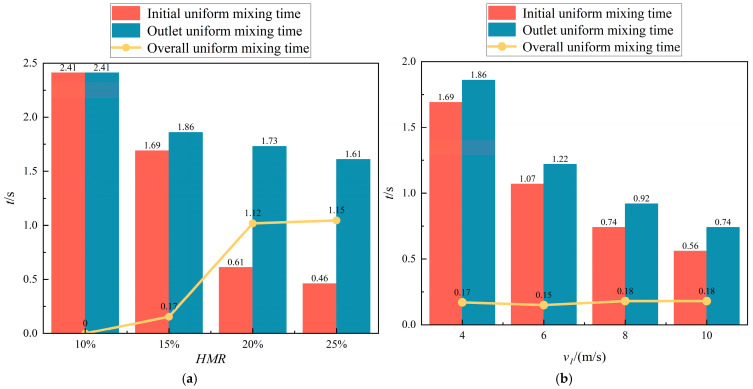
Bar chart comparison of the overall uniform mixing time. (**a**) Variation in HMR; (**b**) Variation in methane flow rate.

Based on the definition of overall uniform mixing time, a comparative investigation was conducted between the uniform mixing time at the pipe outlet and the initial uniform mixing time, with the comparative results illustrated in Figure 33. Combined analysis of Figure 33a,b reveals that while both methods reduce the uniform mixing time, increasing the HMR primarily shortens the initial uniform mixing time in the pipeline, with limited impact on the outlet uniform mixing time. As shown in the figure, the overall uniform mixing time gradually increases with increasing HMRs, while the methane flow rate has minimal impact on it. This is consistent with the research results of the natural gas and hydrogen mixture conducted by Christian Charco (2018) [50], where the total mixing time increases with the increase in hydrogen flow rate and natural gas pipeline diameter. This is because an increase in the HMR results in more hydrogen being introduced into the pipeline. Given the higher diffusivity of hydrogen, the mixing process is accelerated, thereby reducing the required mixing length. However, at the outlet, the dynamic adjustment of gas states becomes more complex due to factors such as residual concentration gradients, which prolongs the overall mixing adjustment time for the two-phase system. Consequently, increasing the HMR optimizes spatial efficiency in mixing (requiring a shorter pipeline length to achieve mixing) but incurs a higher total time cost.

On the other hand, an increase in the methane flow rate has a minimal impact on the overall mixing time but effectively reduces the mixing time at the outlet section. While it does not significantly alter the mixing process within the pipeline, it accelerates the stabilization time of gases at the outlet with consistent effectiveness. Therefore, increasing the methane flow rate offers limited improvement in overall mixing time, but its primary benefit lies in the rapid stabilization at the outlet section. However, this may necessitate longer pipeline lengths (increased uniform mixing length) to compensate for localized disturbances caused by higher flow rates. In other words, variations in hydrogen content significantly affect the uniform mixing time of the hydrogen–methane system within the pipeline, whereas changes in methane flow rate have a negligible impact on the system’s adjustment time.

This study elucidates the influence of the hydrogen-to-methane ratio (HMR) and methane flow rate on the mixing process through spatial and temporal dimensions, quantified by the uniform mixing length (spatial metric) and overall uniform mixing time (temporal metric), respectively. The mechanisms governing these impacts are distinct for HMR and methane flow rate, as outlined below:

(1) Increasing HMR:

Spatial Effect: Elevating the HMR shortens the uniform mixing length (Figure 29a), indicating that the cross-section achieving homogeneity is located closer to the hydrogen injection point.

Temporal Effect: However, a higher HMR reduces mixing kinetics, prolonging the overall uniform mixing time (Figure 33a). Consequently, while a shorter pipeline length is required to achieve uniformity, the operational duration for complete mixing increases.

(2) Increasing Methane Flow Rate:

Spatial Effect: A higher methane flow rate marginally reduces the uniform mixing length, though this effect is less pronounced compared to HMR (Figure 29b).

Temporal Effect: Enhanced flow rates sustain rapid mixing dynamics, significantly shortening the overall uniform mixing time (Figure 33b). Thus, a longer pipeline is necessary to accommodate the accelerated flow, but the time to achieve homogeneity decreases.

The observed time–space trade-off mechanism provides critical insights for optimizing hydrogen-blended natural gas pipeline design. In scenarios where hydrogen injection points deviate from natural gas supply inlets, this mechanism informs strategic decisions:

(1) Cost-Driven Design: To minimize infrastructure costs (shorter mixing lengths), increasing HMR is advantageous, albeit at the expense of longer operational adjustment periods.

(2) Dynamic Response Prioritization: If rapid adaptation to fluctuating demand is critical, elevating methane flow rates reduces mixing time, though necessitating extended pipeline segments to manage higher velocities.

These findings refine design protocols by balancing spatial constraints with operational flexibility, enhancing the feasibility of hydrogen integration into existing gas networks. This compromise mechanism reflects the technical and economic challenges encountered in the Ningdong Energy and Chemical Industry Base project (summarized in Table 1), which focuses on the green hydrogen–coal chemical integration as its core initiative while incorporating hydrogen-blended natural gas, hydrogen mobility, and liquid hydrogen storage/transportation as key demonstration scenarios. Within the hydrogen–natural gas blending framework, four dedicated test pipelines were constructed to evaluate material compatibility (e.g., pipeline steels, valves) under hydrogen blending ratios (HBRs) ranging from 6% to 24%, enabling empirical validation of safety and economic feasibility.

Parametric optimization of hydrogen injection protocols in this project achieves a viable balance between infrastructure capital costs and hydrogen injection strategies. A systematic analysis of techno-economic trade-offs identifies optimal hydrogen blending ratios that reconcile technical constraints with economic viability, providing critical insights for industrial-scale deployment and informing the development of national standards for hydrogen–natural gas co-transportation.

Furthermore, the empirical insights derived from this initiative—particularly the spatial constraints–operational flexibility equilibrium and hydrogen injection positioning strategies—advance the formulation of robust technical protocols. These efforts directly contribute to standardizing design practices and accumulating operational benchmarks for China’s emerging hydrogen-blended pipeline infrastructure.

## 4. Conclusions

This study employs numerical simulation methodologies to conduct transient state simulations of hydrogen-blended natural gas pipelines with T-junction configurations. The research systematically investigates the temporal evolution of hydrogen mole concentration, coefficient of variation (COV), and pressure drop within the pipeline system. Furthermore, it establishes the quantitative relationships between hydrogen mixing rates (HMRs) and methane flow rates, and their impacts on hydrogen concentration distribution, uniform mixing length, and uniform mixing time. The findings provide valuable scientific insights and practical references for the engineering design and operational optimization of hydrogen–natural gas blending systems in industrial applications. The principal conclusions of this research are as follows:

(1) The gas stratification phenomenon in the main pipeline can be categorized into gravitational stratification and radial stratification. Gravitational stratification is primarily driven by the density disparity between methane and hydrogen, where methane exhibits higher density compared to hydrogen. Radial stratification arises from two key factors: when the hydrogen jet-to-methane flow rate ratio exceeds critical thresholds, and the hydrogen jet penetration depth increases significantly. These conditions induce vortex dynamics in the injection zone, causing hydrogen migration from lateral regions to the upper section of the pipeline, thereby creating radial concentration gradients.

(2) The results demonstrate that increasing hydrogen injection volume from 10% to 25% significantly reduces the uniform mixing length from 100.000 *D* to 20.875 *D*. This parametric study reveals that the HMR serves as the predominant control parameter governing mixing efficiency. Concurrently, increasing methane flow rate from 4 m/s to 10 m/s reduces uniform mixing length from 90.875 *D* to 71.500 *D*. Compared with the amount of HMR, the influence of the flow velocity on the mixing distance is weak, but it is easy to operate.

(3) The temporal evolution of the pressure drop reveals three distinct trends: an initial rapid increase, followed by an immediate decrease, and concluding with a gradual reduction until reaching stability. Both HMR and methane flow rate variations influence the pipeline flow dynamics, with increased HMRs and methane flow rates leading to elevated flow rates and corresponding pressure drops. However, the HMR exhibits limited impact on the flow rate, whereas methane flow rate modifications significantly affect the flow rate. Consequently, the pressure drop demonstrates greater sensitivity to variations in methane flow rate compared to hydrogen injection volume.

(4) The coefficient of variation (COV) demonstrates distinct trends along the pipeline length and over time. Along the axial distance, the COV initially decreases rapidly, followed by a slight decrease or marginal increase, eventually reaching a steady decline. Cross-sectionally, the COV exhibits a rapid temporal decrease, subsequently displaying a slight increase before stabilizing at a constant value.

(5) The adjustment of HMR and methane flow rate significantly impacts the mixing dynamics in the pipeline system. Specifically, increasing the HMR primarily accelerates the mixing process in the initial section of the pipeline, while demonstrating minimal effect on the uniform mixing time at the outlet section. Conversely, increasing the methane flow rate shows limited influence on the mixing efficiency in the initial pipeline section, but effectively reduces the mixing time at the outlet section with enhanced stability and reduced fluctuation. A fundamental trade-off in engineering applications is established: adjusting HMR effectively reduces uniform mixing length but increases overall uniform time, while modifying methane flow rate shortens overall uniform mixing time at the expense of increased uniform mixing length.

## Figures and Tables

**Table 1:** Global natural gas–hydrogen blending pipeline projects.

Project Name	Year	Pipeline Pressure (MPa)	H_2_ Blending Ratio (%)	Hydrogen
Baotou–Linhe Gas Pipeline, China	2024	10	10	Integrated digital management system for full-process monitoring.
Hebei Zhangjiakou Pilot, China	2023	-	-	Validated gas appliance compatibility and safety monitoring protocols.
Ningdong Energy Base Project, China	2023	2.0	6–24	Assessed pipeline material compatibility, blending processes, and end-use combustion.
HyDeploy Project, UK [2]	2017–2018	-	20	First European residential hydrogen blending trial; verified grid compatibility.
Energiepark Mainz, Germany [3]	2013	-	10	2 MW pilot plant injecting electrolytic hydrogen into the gas grid.
NATURALHY, EU [4]	2004–2009		20	The impact of natural gas and hydrogen mixing on safety.
Ameland Project, Netherlands [4]	2008–2011	-	12	Utilized non-metallic pipelines for corrosion resistance.
GRHYD Project, France [4]	2014–2018	-	6–20	Supplied blended gas for district heating and hydrogen-fueled public transport.

**Table 2:** Physical properties of pipeline fluid (288.15 K, 0.1 MPa).

Physical Properties	Methane	Hydrogen
Density (kg/m^3^)	0.6679	0.08189
Viscosity (Pa·s)	11.067 × 10^−6^	8.411 × 10^−6^

**Table 3:** Operating parameter settings.

Simulation Scheme	Methane Flow Rate (m/s)	Hydrogen Mass Flow Rate (kg/s)	Turbulence Intensity (%)	HMR (%)
Case 1	4	1.89 × 10^−4^	4.64	10
Case 2	4	2.91 × 10^−4^	4.63	15
Case 3	4	4.12 × 10^−4^	4.61	20
Case 4	4	5.49 × 10^−4^	4.60	25
Case 5	6	4.36 × 10^−4^	4.32	15
Case 6	8	5.81 × 10^−4^	4.24	15
Case 7	10	7.26 × 10^−4^	4.05	15

Note: HMR refers to hydrogen blending ratio.

**Table 4:** The coordinates of the monitoring curves.

Monitoring Curve Number	Origin Coordinates (mm)	Terminal Coordinates (mm)
1	(39, 0, 0)	(39, 8000, 0)
2	(20, 0, 20)	(20, 8000, 20)
3	(20, 0, −20)	(20, 8000, −20)
4	(0, 0, 39)	(0, 8000, 39)
5	(0, 0, 0)	(0, 8000, 0)
6	(0, 0, −39)	(0, 8000, −39)
7	(−20, 0, 20)	(−20, 8000, 20)
8	(−20, 0, −20)	(−20, 8000, −20)
9	(−39, 0, 0)	(−39, 8000, 0)

**Table 5:** Experimental parameters for different operating conditions.

Variable	Design Group	Methane Inlet Flow Rate (m/s)	Hydrogen Blending Ratio (%)
Hydrogen mixing ratio (HMR)	Case 1	4	10
	Case 2	4	15
	Case 3	4	20
	Case 4	4	25
Methane inlet flow rate	Case 2	4	15
	Case 5	6	15
	Case 6	8	15
	Case 7	10	15

## Data Availability

All data generated or analyzed during this study are included in the article.

## Supplementary Materials

The following supporting information can be downloaded at: https://www.mdpi.com/article/10.3390/ma18081879/s1, Figure S1: Temporal evolution of the hydrogen mole concentration at different monitoring cross-sections when changing HMR; Figure S2: Bar chart of uniform mixing time of hydrogen mole fraction at different monitoring sections when changing the HMR.

## Abbreviations

The following abbreviations are used in this manuscript:

HMR	Hydrogen mixing ratio
COV	Coefficient of variation
HHV	Higher heating value
CFD	Computational fluid dynamics
DNN	Deep neural network
CFL	Courant–Friedrichs–Lewy
HPP	Hydrogen partial pressure
HMFV	Hydrogen mole fraction variance
