# Supplementary figures and images for: Numerical Simulation of Hydrogen Mixing Process in T-Junction Natural Gas Pipeline
Source: Materials (Basel). 2025 Apr 20;18(8):1879. doi: 10.3390/ma18081879 (PMC12029052; doi:10.3390/ma18081879)

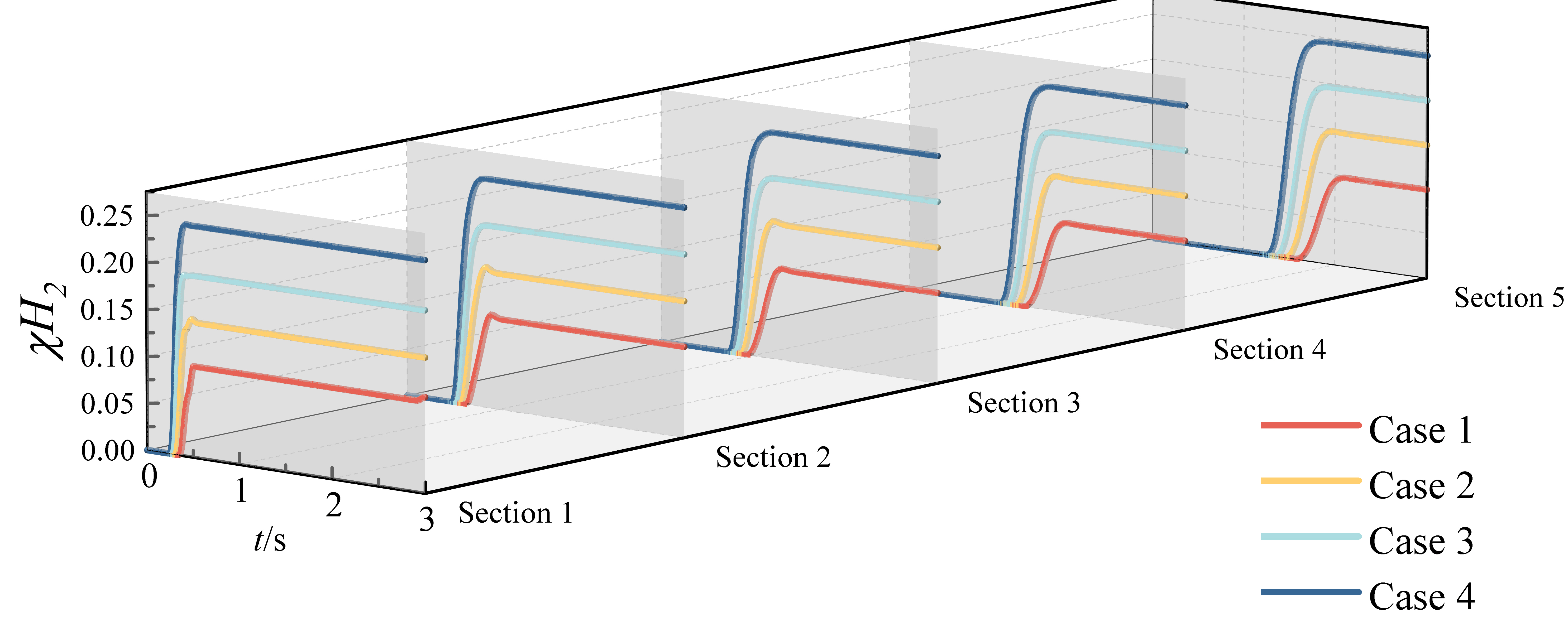

Supplement: Supplementary file 1 [file materials-18-01879-s001.zip › Supplementary File/Figure S1 Temporal evolution of the hydrogen mole concentration at different monitoring cross-sections when changing HMR.jpg]

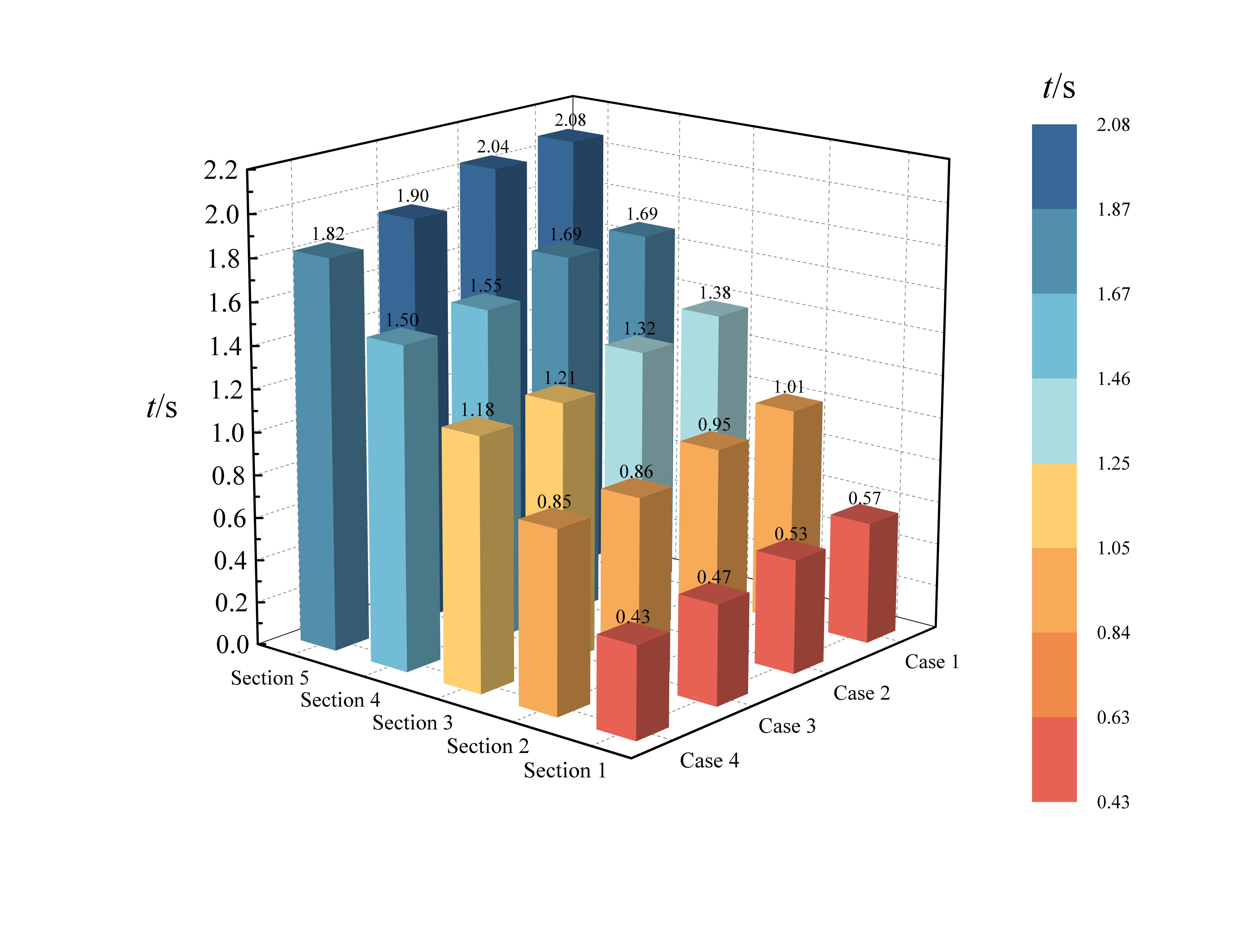

Supplement: Supplementary file 1 [file materials-18-01879-s001.zip › Supplementary File/Figure S2 Bar chart of uniform mixing time of hydrogen mole fraction at different monitoring sections when changing the HMR.jpg]
